# Methanogenic archaea use a bacteria-like methyltransferase system to demethoxylate aromatic compounds

**DOI:** 10.1038/s41396-021-01025-6

**Published:** 2021-06-18

**Authors:** Julia M. Kurth, Masaru K. Nobu, Hideyuki Tamaki, Nadieh de Jonge, Stefanie Berger, Mike S. M. Jetten, Kyosuke Yamamoto, Daisuke Mayumi, Susumu Sakata, Liping Bai, Lei Cheng, Jeppe Lund Nielsen, Yoichi Kamagata, Tristan Wagner, Cornelia U. Welte

**Affiliations:** 1 Department of Microbiology, Institute for Water and Wetland Research, Radboud University, Nijmegen, The Netherlands; 2 Soehngen Institute of Anaerobic Microbiology, Radboud University, Nijmegen, The Netherlands; 3 Bioproduction Research Institute, National Institute of Advanced Industrial Science and Technology (AIST), Tsukuba, Japan; 4 Department of Chemistry and Bioscience, Aalborg University, Aalborg East, Denmark; 5 Netherlands Earth System Science Center, Utrecht University, Utrecht, The Netherlands; 6 Bioproduction Research Institute, National Institute of Advanced Industrial Science and Technology (AIST), Sapporo, Japan; 7 Institute for Geo-Resources and Environment, Geological Survey of Japan, National Institute of Advanced Industrial Science and Technology (AIST), Tsukuba, Japan; 8 Key Laboratory of Energy Microbiology and Its Application of Ministry of Agriculture, Biogas Institute of Ministry of Agriculture, Chengdu, China; 9 Microbial Metabolism research group, Max Planck Institute for Marine Microbiology, Bremen, Germany

**Keywords:** Archaeal physiology, Soil microbiology, Proteomics

## Abstract

Methane-generating archaea drive the final step in anaerobic organic compound mineralization and dictate the carbon flow of Earth’s diverse anoxic ecosystems in the absence of inorganic electron acceptors. Although such *Archaea* were presumed to be restricted to life on simple compounds like hydrogen (H_2_), acetate or methanol, an archaeon, *Methermicoccus shengliensis*, was recently found to convert methoxylated aromatic compounds to methane. Methoxylated aromatic compounds are important components of lignin and coal, and are present in most subsurface sediments. Despite the novelty of such a methoxydotrophic archaeon its metabolism has not yet been explored. In this study, transcriptomics and proteomics reveal that under methoxydotrophic growth *M. shengliensis* expresses an *O*-demethylation/methyltransferase system related to the one used by acetogenic bacteria. Enzymatic assays provide evidence for a two step-mechanisms in which the methyl-group from the methoxy compound is (1) transferred on cobalamin and (2) further transferred on the C_1_-carrier tetrahydromethanopterin, a mechanism distinct from conventional methanogenic methyl-transfer systems which use coenzyme M as final acceptor. We further hypothesize that this likely leads to an atypical use of the methanogenesis pathway that derives cellular energy from methyl transfer (Mtr) rather than electron transfer (F_420_H_2_ re-oxidation) as found for methylotrophic methanogenesis.

## Introduction

Methanogenesis evolved more than 3.46 Gyr ago and has profoundly contributed to Earth’s climate [1, 2]. About 70% of the emitted methane (CH_4_) is produced by methane-generating archaea (methanogens; [3]) underlining the importance of methanogenesis for the global carbon cycle. Methanogens are known to produce methane from one- to two-carbon substrates (i.e., carbon dioxide [CO_2_], acetate, and methylated compounds), often using (in)organic compounds as electron donors (e.g., hydrogen [H_2_] and formate). Three major pathways of methanogenesis are known. In the hydrogenotrophic pathway, H_2_ (or formate) are used as electron donors with carbon dioxide as electron acceptor. In the methylotrophic pathway, small methylated carbon compounds are converted to methane and carbon dioxide. In the aceticlastic pathway, acetate is cleaved to methane and carbon dioxide [4]. Beyond this, a thermophilic methanogen isolated from a deep subsurface environment [5], *Methermicoccus shengliensis*, was recently discovered to directly generate methane from a variety of methoxylated aromatic compounds (ArOCH_3_) [6]. Methoxylated aromatic compounds are derived from lignin and occur in large quantities on Earth [7]. The environmental abundance of methoxylated aromatics indicates that methoxydotrophic archaea might play a so far unrecognized and underestimated role in methane formation and carbon cycling of coal, lignin, and other humic substances, especially in the subsurface [8]. Aromatic compounds are a major component of crude oil with about 20–43% [9, 10], and it is quite likely that methoxylated aromatic compounds in oil might be degraded by methoxydotrophic organisms. As *M. shengliensis* has been isolated from oil production water [5], the organism might play a role in the degradation of methoxy compounds in oil reservoirs. Next to oil, methoxylated aromatic compounds are components of coal. Although conversion of coal compounds to methane has been thought to require metabolic interactions [11], *Methermicoccus*’ ability to accomplish this alone might have significant implications for coalbed methane formation (7% of global annual methane formation [12]), including enhanced methane recovery [13]. Therefore, it is important to understand the unique methoxy compound-degrading methane-forming metabolism of *M. shengliensis*.

The discovery of the methoxydotrophic ability of *M. shengliensis* revealed that the capacity to degrade methoxylated aromatic compounds is not confined to bacteria as previously thought, yet how *M. shengliensis* (and thus archaea) accomplish methoxydotrophic methanogenesis remains unknown. The organism is also capable of and possesses the necessary genes for methylotrophic methanogenesis [6], in which a methylated substrate (e.g., methanol) is disproportionated to ¾ CH_4_ and ¼ CO_2_. In principle, degradation of methoxy groups could follow a similar pathway, given that methyl and methoxy groups have the same oxidation state. However, isotope-based investigation showed that methoxydotrophic methanogenesis unprecedentedly entails both methyl disproportionation and CO_2_ reduction to CH_4_ [6], suggesting the involvement of a novel methanogenic pathway. In this study, integration of genomics, transcriptomics, and proteomics reveals that *M. shengliensis* methoxydotrophy employs a novel methyltransferase system for ArOCH_3_ *O*-demethylation. While known methanogens transfer methyl compounds using coenzyme M (CoM) as a C_1_ carrier [14], we suggest that the *M. shengliensis* ArOCH_3_ methyltransferase rather uses tetrahydromethanopterin (H_4_MPT) as final C_1_ carrier. The different entry point into methanogenesis (i.e., as CH_3_-H_4_MPT rather than CH_3_-CoM) putatively prompts changes in energetics, thermodynamics, and kinetics that might involve an idiosyncratic C_1_ catabolism cycling between oxidation and reduction.

## Materials and methods

### Genome analysis

The functions of individual proteins were predicted using homology, phylogeny, and domain identification. Homology of *M. shengliensis* proteins with proteins in reference genomes was calculated using NCBI blastp. For each protein, phylogenetic analysis was performed by collecting homologues from the SwissProt and UniProt databases [15], protein sequence alignment using MAFFT v7.394 [16], and phylogenetic tree construction using RAxML-NG v0.5.1b [17] or FastTree v 2.2.11 [18]. Annotations were verified using domain-based function annotation involving NCBI CD-SEARCH/CDD v3.18 (10.1093/nar/gku1221), InterProScan v5 [19], SignalP 4.1 [20], and Prosite (https://prosite.expasy.org).

### Cultivation of Methermicoccus shengliensis

*M. shengliensis* AmaM was cultivated as described previously [6]. The following medium was used for the AmaM cultures: a bicarbonate-buffered mineral medium (pH 7.0) [21] containing 0.15 g l^−1^ KH_2_PO_4_, 0.5 g l^−1^ NH_4_Cl, 0.2 g l^−1^ MgCl_2_·6H_2_O, 0.15 g l^−1^ CaCl_2_·2H_2_Og, 2.5 g l^−1^ NaHCO_3_, 0.3 g l^−1^ cysteine·HCl, 0.3 g l^−1^ Na_2_S·9H_2_O, 20.5 g l^−1^ NaCl, 1 ml trace elements solution (DSMZ medium 318 with slight modifications: NaCl was eliminated, and 3 mg l^−1^ of Na_2_WO_4_·2H_2_O and 2 mg l^−1^ of Na_2_SeO_3_ were added), 1 ml vitamin solution (DSMZ medium 141 with a slight modification: all components were mixed at a concentration of 20 μmol l^−1^), and 1 ml resazurin solution (1 mg ml^−1^). As an energy source, cultures were grown with either methanol (MeOH) (10 mM), trimethylamine (10 mM), 2-methoxybenzoate (10 mM), or 3,4,5-trimethoxybenzoate (TMB) (10 mM). A 0.5 M stock solution of TMB was produced by dissolving 3,4,5-trimethoxybenzoic acid in water and adjusting the pH to 7 with NaOH.

*Methermicoccus shengliensis* ZC-1 (DSM 18856) was obtained from the DSMZ (Braunschweig, Germany) and cultivated in modified DSM medium 1084. Sludge fluid was replaced by trace element solution (100 x trace element solution: 1.5 g l^−1^ nitrilotriacetic acid, 3 g l^−1^ MgSO_4_·7 H_2_O, 0.45 g l^−1^ MnSO_4_·2 H_2_O, 1 g l^−1^ NaCl, 0.1 g l^−1^ FeSO_4_·7 H_2_O, 0.18 g l^−1^ CoSO_4_·6 H_2_O, 0.1 g l^−1^ CaCl_2_·2 H_2_O, 0.18 g l^−1^ ZnSO_4_·7 H_2_O, 0.01 g l^−1^ CuSO_4_·5 H_2_O, 0.02 g l^−1^ KAl(SO_4_)_2_·12 H_2_O, 0.01 g l^−1^ H_3_BO_3_, 0.01 g l^−1^ Na_2_WO_4_·2 H_2_O, 0.01 g l^−1^ Na_2_MoO_4_·2 H_2_O, 0.025 g l^−1^ NiCl_2_·6 H_2_O, 0.01 g l^−1^ Na_2_SeO_3_) and vitamin solution (1000 x vitamin solution: 20 mg l^−1^ biotin, 20 mg l^−1^ folic acid, 100 mg l^−1^ pyridoxine-HCl, 50 mg l^−1^ thiamin-HCl·2 H_2_O, 50 mg l^−1^ riboflavin, 50 mg l^−1^ nicotinic acid, 50 mg l^−1^ D-Ca-pantothenate, 2 mg l^−1^ vitamin B_12_, 50 mg l^−1^ p-aminobenzoic acid, 50 mg l^−1^ lipoic acid). The amount of supplied coenzyme M was reduced 20-fold (0.13 g l^−1^) and 2.5 g l^−1^ NaHCO_3_ instead of 1 g l^−1^ Na_2_CO_3_ was used. The medium was sparged with N_2_:CO_2_ in a 80:20 ratio before autoclaving. As substrate either 150 mM MeOH or 10 mM TMB were used. The cultures were incubated at 65 °C. Identity of the organism was checked by 16 S rRNA gene sequencing of DNA from TMB grown cell with primers Arch349F (5′-GYGCAGCAGGCGCGAAA-3′) and Arch806R (5′-GGACTACVSGGGTATCTAAT-3′) [22].

### RNA isolation from *M. shengliensis* cells and sequencing

For transcriptomics, *M. shengliensis* AmaM was cultivated in 130-ml serum vials containing 50 ml of the medium with 10 mM MeOH, 2-methoxybenzoate, trimethylamine, or TMB as the sole organic carbon substrate. Total RNA was extracted from the cells harvested in the exponential growth phase through brief centrifugation (3 min at 9000 × *g* at room temperature) using methods described by Schmidt et al. [23] with slight modifications. In brief, after adding extraction buffer (0.1 M Tris-HCl, 0.1 M ethylenediaminetetraacetic acid, 0.75 M sucrose), cells were enzymatically and chemically lysed by lysozyme (1 mg ml^−1^), achromopeptidase (0.01 mg ml^−1^), proteinase K (0.1 mg ml^−1^), and sodium dodecyl sulfate (1% [w/v]). The nucleic acid fraction was extracted using cetyl trimethyl ammonium bromide (1% [w/v]) and chloroform-isoamyl alcohol (24:1). Extracted nucleic acids were precipitated with isopropanol and washed with ethanol, and then fractionated into DNA and RNA by ALLPrep DNA/RNA mini kit (Qiagen, Hilden, Germany), according to manufacturer’s instructions. RNA samples were treated with DNase to remove DNA contaminants. Removal of DNA contamination from the samples was confirmed by PCR amplification. RNA concentrations were measured using a Nanodrop 2000c and Qubit Fluorometer using Qubit RNA HS (Thermo Fisher Scientific, Wilmington, DE, USA).

The resulting cDNA was fragmented using Bioruptor (Diagenode, Inc., Denville, NJ USA), profiled using Agilent Tapestation, and subjected to Beckman Biomek FXp (Biomek 6000, Beckman Coulter) fully automatic workstation and a Beckman HT library kit (SPRIworks HT, Beckman Coulter, Inc. CA USA; PN B09855AA) to generate fragment libraries. The instructions were strictly followed to perform library construction. Briefly, after fragmentation the ends were repaired and the fragments were subsequently adenylated. Adapters were then ligated to both ends. The adaptor-ligated templates were further purified using Agencourt AMPure SPRI beads (Beckman Coulter, Inc. CA USA). The adaptor-ligated library was amplified by ligation-mediated PCR which consisted of 11 cycles of amplification, and the PCR product was purified using Agencourt AMPure SPRI beads again. After the library construction procedure was completed, QC was performed using Nanodrop 2000 (Thermo Scientific, USA) and an Agilent TapeStation (Agilent, USA) to ensure the library quality and quantity. Alternatively, cDNA was profiled using Agilent Bioanalyzer, and subjected to library preparation using NEBNext reagents (New England Biolabs, Ipswich, MA USA, catalog# E6040). The quality and quantity and size distribution of the libraries were determined using an Agilent Bioanalyzer 2100.

Sequencing was performed on the HiSeq 2500 (Rapid run, Illumina, CA USA) with chemistry v3.0 and using the 2 × 100 bp paired-end read mode and original chemistry from Illumina according to the manufacturer’s instructions. The initial data analysis was started directly on the HiSeq 2500 System during the run. The HiSeq Control Software 2.0.5 in combination with RTA 1.17.20.0 (real-time analysis) performed the initial image analysis and base calling. In addition, CASAVA-1.8.2 generated and reported run statistics and the final FASTQ files comprising the sequence information which was used for all subsequent bioinformatics analyses. Sequences were de-multiplexed according to the 6 bp index code with 1 mismatch allowed. Alternatively, the libraries were then submitted for Illumina HiSeq2000 sequencing according to the standard operation. Paired-end 90 or 100 nucleotide (nt) reads were generated, checked for data quality using FASTQC (Babraham Institute, Cambridge, UK).

For *M. shengliensis* ZC-1, cells were harvested in the exponential phase (MeOH grown cells: OD_600_ 0.150 to 0.240 and TMB grown cells: OD_600_ 0.110 to 0.180) at 10,000 × *g*, 25 min and 4 °C. The pellet was frozen in liquid nitrogen and stored at −80 °C until RNA isolation. RNA isolation was performed with the RiboPure-Bacteria Kit (Thermo Fischer Scientific) according to manufacturer’s instructions. Quantity and quality of RNA from MeOH and TMB grown cells (in triplicates) was checked with an Agilent 2100 Bioanalyzer and the RNA Integrity Number was between 7.2 and 8.2.

For library preparation the TruSeq Stranded mRNA Library Prep protocol (Illumina, San Diego, California USA) was used according to the manufacturer’s instructions. Total RNA was used for library preparation. The library concentration measured with a Qubit fluorometer and the average fragment size obtained with the Agilent 2100 Bioanalyzer were used to calculate the correct dilution factor required for normalization of the library. After dilution to 4 nM and denaturation using the Denature and Dilute Libraries Guide (Illumina, San Diego, CA), the library was sequenced using a MiSeq machine (Illumina, San Diego, CA) to generate 150 bp single-end reads.

To analyze the AmaM and ZC-1 transcriptomic data, raw reads from the MiSeq platform were trimmed using Trimmomatic v0.36 (SLIDINGWINDOW:6:30 MINLEN:50 LEADING:3 TRAILING:3) [24] and mapped to the respective genomes (AmaM – JGI IMG/M ID 2516653088; ZC-1 – GenBank accession number NZ_JONQ00000000.1) using BBMap v38.26 (semiperfectmode = t) (https://jgi.doe.gov/data-and-tools/bbtools/).

### Analysis of *M. shengliensis* ZC-1 whole cell proteome

After cultivation of *M. shengliensis* in medium with either MeOH or TMB as substrate cells were harvested anaerobically (13,000 × *g*, 25 min) in the exponential phase (OD_600nm_ 0.2–0.4), pellets were frozen in liquid nitrogen and freeze-dried before they were stored at −80 °C in quadruplicates.

After adding 400 µL ammonium bicarbonate (100 mM, pH 8) and resuspending, the samples were transferred together with 300 µL TEAB resuspension buffer (50 mM triethylammonium bicarbonate, 1% (w/w) sodium deoxycholate, pH 8.0) and 300 µL B-PER reagent (Thermo Fisher) to 200 mg glass beads in shock resistant 2 mL tubes. Bead beating was performed using Precellys 24 (Bertin Technologies, France) at 6000 rpm for 20 s with a 30 s break for three cycles. After centrifugation at 14,000  × *g* for 10 min at 4 °C, supernatant was transferred to a 1.5 mL Eppendorf tube.

To precipitate the proteins one volume 100% TCA was added to four volumes of protein extract. After incubation at 4 °C for 10 min, the supernatant was removed and the pellet washed two times with 200 µL ice cold acetone (centrifugation at 14,000 ×  *g* for 5 min at 4 °C). The pellet was dried at 95 °C for 5 min and resuspended in 50 µL ammonium bicarbonate (100 mM, pH 8). Protein concentration was estimated using Qubit Protein Assay Kit (Thermo Fisher Scientific) and a Qubit 3.0 fluorometer (Thermo Fisher Scientific).

In solution digestion was performed by adding one volume TEAB resuspension buffer to the protein extracts followed by incubation at 99 °C for 5 min. Subsequently, samples were reduced using 1 µg TCEP per 25 µg protein and incubation at 37 °C for 30 min, and alkylated with 1 µg Iodoacetamide per 10 µg protein followed by incubation at 37 °C for 20 min in the dark. Digestion was performed using 1 µg trypsin per 50 µg protein and incubation at 37 °C for 16 h. A final concentration of 2% formic acid was added to the samples and after 5 min incubation at room temperature, the samples were centrifuged at 13,000 × *g* for 20 min at 4 °C. The supernatant was transferred into a new tube.

Samples were desalted using a modified StageTIP protocol [25] and subsequently lyophilised in a SpeedVac centrifuge. Peptides were reconstituted in 2% (v/v) acetonitrile and 0.1% formic acid prior to analysis.

LC-MS/MS analysis of the samples was performed using an Easy-nLC 1200 system (Thermo Scientific) coupled to a Q Exactive HF mass spectrometer (Thermo Scientific) through a Nanospray Flex ion source (Thermo Scientific). Peptides were loaded onto an Acclaim PepMap 100 (100 μm × 2 cm, NanoViper, C18, 5 μm, 100 A) (Thermo Scientific) trap column and separated on an analytical column at a flow rate of 300 nL min^−1^, during a 40 min linear gradient, ranging from 0 to 100% of a mobile phase containing acetonitrile.

Mass spectrometry was performed in positive mode only, fragmenting precursors with an assigned charge of ≥2. An isolation window of 1.2 *m/z* was used and survey scans were acquired at 400–1200 *m/z* at resolution 60,000 at *m/z* 200, and fragmentation spectra were captured at 15,000 at *m/z* 200. Maximum ion injection time was set to 50 ms for MS and 45 ms for MS/MS scans. Automatic gain for survey scans was set to 1e6 ions and 1e5 ions for fragmentation scans. The apex trigger was not set, the intensity threshold was set to 4.4e4 ions and dynamic exclusion of 30 s was applied. Normalized Collision Energy was set to 28, “peptide match” was set to “preferred” and “exclude isotopes” was enabled.

Q-exactive RAW data files were processed using MaxQuant (v1.6.3.4) [26], with carbamidomethylation set as a fixed modification and methionine oxidation as a variable modification, a protein, and peptide false discovery rate (FDR) of 1% and label-free quantification (LFQ) as implemented in MaxQuant. Data were searched against a database consisting of the predicted open reading frames (ORFs) of the draft genome of *M. shengliensis* ZC-1 (NZ_JONQ00000000.1), as well as the ORFs of closely related organisms *Methanothrix thermoacetophila* (UP000000674), *Methanothrix harundinacea* (UP000005877), *Methanosarcina barkeri* (UP000033066), *Methanolacinia petrolearia* (UP000006565) and *Methanomethylovorans hollandica* (UP000010866), downloaded from UniProt on 18-01-2019.

Data analysis was performed using Perseus (v1.6.2.3) [27]. Student’s *t* test was performed using a significance level of *p* ≤ 0.05 and permutation-based FDR at 5%. The relative protein abundances were represented as Log2-transformed LFQ values. Fold change was expressed as the ratio of averaged LFQ value of a protein across all replications of *M. shengliensis* fed with TMB divided by the averaged LFQ value of those fed with methanol.

### Native purification of MtoA and MtoB from *M. shengliensis*

All steps were performed under an anaerobic atmosphere and all buffers were prepared anaerobically. About 6 g (wet weight) of *M. shengliensis* ZC-1 cells harvested in the late exponential phase were defrosted while gassing for 10 min with N_2_ gas and passed in an anaerobic tent containing an atmosphere of N_2_/CO_2_ at a ratio of 90:10%. Afterwards, cells were resuspended in 20 mL anaerobic IECA buffer (50 mM Tris/HCl pH 8, 2 mM dithiothreitol abbreviated as DTT), sonicated (Bandelin sonopuls 6 × 50 % power for 10 s with 20 s break) and centrifuged (13,000 × *g*, 30 min at room temperature) to remove cell debris. The supernatant was collected and the pellets were resuspended in 10 ml anaerobic IECA buffer, sonicated (5 × 50 % power for 10 s with 20 s break), centrifuged (13,000 × *g*, 30 min) and the supernatant combined with the supernatant from the previous step. The supernatant was anaerobically transferred to a Coy tent containing a gas atmosphere of N_2_/H_2_ at a ratio of 97:3% and was then diluted fourfold with IECA buffer, filtered through 0.2 μm filters (Sartorius), and loaded on a 20 ml DEAE FF column equilibrated with IECA buffer (GE healthcare). Proteins were eluted by applying a 0 to 40% gradient of 1 M NaCl by using IECB buffer (50 mM Tris/HCl pH 8, 2 mM DTT, 1 M NaCl), over 150 min at a flow rate of 2 ml/min collecting 4 ml fractions. MtoA and MtoB containing fractions were pooled based on sodium dodecyl sulfate–polyacrylamide gel electrophoresis (SDS PAGE) profile. MtoA eluted between 130 and 171 mM NaCl and MtoB between 171 and 200 mM NaCl. Those two pools were diluted 4-fold with HICB (25 mM Tris/HCl pH 7.6, 2 mM DTT, and 2 M (NH_4_)_2_SO_4_), filtered through 0.2 μm filters, and loaded on a 5 ml Phenyl sepharose HP column, separately (GE healthcare). Proteins were eluted by applying a 60 to 0% gradient of 2 M (NH_4_)_2_SO_4_ by using HICA buffer (HICA: 25 mM Tris/HCl pH 7.6, 2 mM DTT) over 60 min at a flow of 1 ml/min collecting 2 ml fractions. MtoA eluted in the range of 1.13 and 0.74 M (NH_4_)_2_SO_4_ and MtoB eluted in the range of 0.45 and 0.10 M (NH_4_)_2_SO_4_. Pooled fractions were diluted 4-fold with HICB, filtered through 0.2 μm filters, and loaded on a Source 15 Phe 4.6/100 PE column (GE healthcare), separately. Proteins were eluted by applying a 70 to 0% gradient of 2 M (NH_4_)_2_SO_4_ by using HICA buffer over 60 min with a flow rate of 1 ml/min collecting 2 ml fractions. Under these conditions MtoA eluted in the range of 1.44 and 1.18 M (NH_4_)_2_SO_4_ and MtoB eluted in the range of 1.27 and 1.09 M (NH_4_)_2_SO_4_. Buffer was exchanged to storage buffer (25 mM Tris/HCl pH 7.6, 10% v/v glycerol, and 2 mM DTT) by a 100–fold dilution using a 15 ml Millipore Ultra-10 centrifugal filter units (Merck; 10 kDa cut-off). Protein concentration was measured by Bradford Protein Assay (Bio-Rad) according to the manufacturer’s instructions.

MtoA and MtoB were identified with help of matrix-assisted laser desorption/ionization time-of-flight mass spectrometry (MALDI-TOF MS) which was performed as explained in the following. Protein bands were cut into small pieces (about 3 × 3 mm) and transferred into an Eppendorf tube. For destaining of the gel pieces, the following solvents/buffers were added successively: 20 µl acetonitrile, 20 µl 50 mM ammonium bicarbonate (ABC) buffer, 50% acetonitrile in ABC buffer and 20 µl acetonitrile. After each addition, samples were swirled and incubated for 10 min at room temperature followed by removing the liquid from the sample. Those steps were repeated, starting from the addition of ABC buffer, until the gel pieces were completely destained. For reduction and alkylation, samples were incubated in 20 µl 10 mM dithiothreitol at 56 °C for 30 min and after removing the liquid from the samples the following solvents/buffers were added successively: 20 µl acetonitrile, 20 µl 50 mM 2-chloroacetamide in 50 mM ABC buffer, 20 µl acetonitrile, 20 µl ABC buffer, 20 µl acetonitrile and 20 µl ABC buffer. After each addition, samples were swirled and incubated for 10 min at room temperature followed by removing the liquid from the sample. For trypsin digestion, 10 µl of 5 ng/µl trypsin in ABC buffer were added to the gel pieces followed by 30 min incubation at room temperature. Afterwards 20 µl ABC buffer were added and the samples were incubated over-night at 37 °C. The samples were sonicated for 20 s in a Branson 2510 sonication bath (Branson, U.S.). Twenty microliters of 0.1% trifluoroacetic acid were added. The samples were swirled and incubated for 20 min at room temperature before the extract liquid was transferred to a new tube. Twenty microliters of acetonitrile were added to the remaining trypsin digest, the samples were swirled and incubated for 30 min at room temperature before the extract liquid was combined with the extract liquid from before. The samples were then dried in a Sanvant ISS110 speedVac (Thermo Scientific) until ~5 µl remain. 0.5 µl of the extracted peptides was pipetted on a MALDI-TOF sample plate and directly mixed with an equal volume of matrix solution containing 10 mg/ml α-cyano-4-hydroxy-α-cyanocinnamic acid in 50% acetonitrile/0.05% trifluoroacetic acid. After drying of the sample this process was repeated once more. A spectrum in the range of 600–3000 *m/z* was recorded using a Microflex LRF MALDI-TOF (Bruker). The Biotools software (Bruker Life Sciences) was used to perform a MASCOT search (Matrix Science Ltd, London, UK) by using the *M. shengliensis* protein database (GenBank accession number NZ_JONQ00000000.1). Search parameters allowed a mass deviation of 0.3 Da, one miscleavage, a variable modification of oxidized methionines and a fixed modification of carbamidomethylated cysteines. For MtoA the molecular weight search (MOWSE) score was 115 and the coverage 41% and for MtoB the MOWSE score was 70 and the coverage 34%.

### Heterologous protein production of MtoC and MtoD

The gene encoding the corrinoid protein MtoC (BP07_RS03260) and the corrinoid activating enzyme (BP07_RS03235) were amplified from genomic *M. shengliensis* DNA with primers 3235fw/3235Srev (CTCATATGAGCGTCAGAGTAACGTTCGAGC, CTGCGGCCGCTTATTTTTCGAACTGCGGGTGGCTCCAGCTAGCTGAAGAGAGTTTTTCTCC) and 3260fw/3260Srev (CTCATATGACGGACGTAAGAGAAGAGCTC/CTGCGGCCGCTTATTTTTCGAACTGCGGGTGGCTCCAGCTAGCCTCCACCCCCACCAGAGC) for cloning in expression vector pET-30a inserting an N-terminal Strep tag via the reverse primer. For cloning of the above mentioned genes into pET-30a (Novagen), primers included NdeI and NotI restriction sites to insert the digested PCR products into the plasmid. PCR was performed with Phusion polymerase (NEB) according to manufacturer’s instructions. For restriction, digest fast digest enzymes (Thermo) were used and for ligation T4-DNA ligase (Promega). *E. coli* DH5α (NEB) was used for plasmid transformation.

For production of the corrinoid protein MtoC (BP07_RS03260) and the corrinoid activating enzyme (BP07_RS03235) the plasmids pET-30a_BP07_RS03260 and pET-30a_BP07_RS03235 were used for transformation into *E. coli* Bl21 (DE3). For protein overexpression, one colony was inoculated in 600 ml LB-medium containing 50 μg/ml kanamycin and incubated at 37 °C and 180 rpm for 16 h. Cells were harvested by centrifugation (15,000 × *g* for 10 min at 4 °C). All further steps were performed anaerobically in an anaerobic hood with anoxic buffers and solutions. Pelleted cells were resuspended in 100 mM Tris-HCl buffer pH 8 containing 150 mM NaCl and lysed by sonication (1 s pulse, 5 s pause, 40% amplitude; 5 min). After removal of insoluble cell material by centrifugation (20,000 × g for 25 min at 4 °C) proteins were purified by Strep-Tactin XT high capacity affinity chromatography according to the manufacturer’s instructions (IBA, Göttingen, Germany). For assessment of purity, sodium dodecyl sulfate-poly-acrylamide gel electrophoresis (SDS-PAGE) was performed.

The protocol for reconstitution of MtoC with cobalamin was adapted from Schilhabel et al. [28]. 1.5 ml (40 mg) anaerobic protein solution was added to 65 ml refolding solution and 650 µl 1 M DTT in a 120 ml glass bottle with a stirrer bar, closed with a rubber stopper (all solutions were made anaerobic by sparging 10 min with nitrogen gas). The refolding solution contained 50 mM Tris, 3.5 M betaine HCl and 1 mM hydroxocobalamin HCl, and pH was adjusted to 7.5. The protein solution then was incubated for 16 h at 4 °C in the dark during slight stirring. Afterwards the buffer was exchanged by Tris HCl pH 7.5 and 1 mM DTT by use of 5 kDa concentration units (Amicon Ultra-15 Centrifugal Filter Units, Merck) several times, until the cobalt containing permeate appeared visibly clear instead of red. Protein was stored anaerobically in 2 ml glass vials closed with air-tight rubber stoppers.

### Enzyme activity assays

Enzyme activity assays were performed in anaerobic 400 µl Quartz cuvettes (number 115-10-40, Hellma) which were closed with a rubber stopper and gassed with N_2_. Cuvettes were heated up to 60 °C before starting the measurements. All measurements were at least performed in triplicates. Anoxic buffers and solutions were added with gas-tight glass syringes (Hamilton, Reno, NE). MtoB activity was determined in a total volume of 300 µl containing a 35 mM Tris HCl, 70 mM KCl, pH 7.5 buffer. Firstly, reconstituted Co(II)-MtoC at 1.2 mg/ml final concentration (about 55 µM) was activated by adding 12 mM MgCl_2_, 0.5 mM Ti(III)citrate (freshly prepared), 2.3 mM ATP and 0.08 mg/ml MtoD. The conversion to Co(I)-MtoC was followed by the change in absorbance at 387 nm on a Cary 60 UV–Vis spectrophotometer (Agilent Technologies, USA) (∆ε_386_ = 21 mM^−1^ cm^−1^ [29] was used for our calculations). The reaction was started by the addition of 2.3 mM 2-methoxybenzoate or TMB and MtoB at a final concentration of 0.015 mg/ml. Fifty microliters of sample were removed before addition of MtoB and after the activity assay for analysis of methoxy compounds by HPLC (see below). Formation of CH_3_-Co(III)-MtoC from Co(I)-MtoC results in a decrease in absorption at 387 nm which was followed with a Cary 60 UV–Vis spectrophotometer (Agilent Technologies, USA). Activity was at least measured in triplicates per substrate. As negative control 2.3 mM methanol or trimethylamine were used.

MtoA activity was determined in a total volume of 300 µl containing a 35 mM Tris/HCl, 70 mM KCl, pH 7.5 buffer. Reconstituted Co(II)-MtoC at 0.4 mg/ml final concentration was activated by adding 0.5 mM Ti(III)citrate (freshly prepared), 2.3 mM ATP and 0.08 mg/ml MtoD. Afterwards 2.3 mM 2-methoxybenzoate and 0.4 mg/ml MtoB were added. As potential methyl group acceptor either 0.8 mM H_4_F (Schircks Laboratories, Switzerland) or 1.7 mM CoM were used. The activity assay was started by addition of 0.03 mg/ml MtoA. Formation of Co(I)-MtoC from CH_3_-Co(III)-MtoC results in a decrease in absorption at 520 nm which was followed with the UV–vis-spectrophotometer.

For illustration of the whole *O*-demethylation/methyl transfer process, UV–vis spectra were recorded from 250–650 nm under the latter described conditions after the sequential addition of MtoC, MtoD plus Ti(III) citrate plus ATP, 2-methoxybenzoate plus MtoB, H_4_F and MtoA.

To measure concentrations of 2-methoxybenzoate and TMB by HPLC an Agilent 1100 HPLC system equipped with a diode array detector (detecting wavelength 230 nm) and a Merck C-18e column (250 mm × 4.6 mm, 5 µm particle size) was used. The flow rate was 0.75 ml/min and a linear gradient was applied: 75% trifluoroacetic acid (TFA; 0.1% in water), 25% acetonitrile to 50% TFA (0.1% in water), 50% acetonitrile in 15 min. Solutions of TMB, 3-OH-4,5-dimethoxybenzoate, 4-OH-3,5-dimethoxybenzoate, 2-methoxybenzoate and 2-hydroxybenzoate in water (0.1 mg/ml) were used as standards. Twenty microliters of sample was used for injection.

### Thermodynamics analyses

Gibbs free energy yield (∆G) was calculated assuming a temperature of 60 °C, pH of 7, CO_2_(g) of 0.2 bar, CH_4_(g) of 0.2 bar, 1 mM NH_4_^+^, and 10 mM for all other compounds. Temperature adjustments were made as described previously [30]. For methoxylated compounds (2-methoxybenzoate and 3,4,5-trimethoxybenzoate), Gibbs free energy of formation (∆G_f_) and enthalpy of formation (∆H_f_) were first estimated for the acid form using the Joback group contribution method [31]. After calculating the ∆G_f_ at 60 °C using the Van’t Hoff equation, the ∆G_f_ of the carboxylate forms at 60 °C were then calculated using pK_a_ estimated by ChemAxon Marvin (https://chemaxon.com/products/marvin). The ∆H_f_ of the carboxylate forms were estimated using ∆G_f_ at 25 and 60 °C and the Van’t Hoff equation. The ∆G for methyl transfer from 2-methoxybenzoate, methanol, and methylamine to tetrahydromethanopterin or coenzyme M was calculated by subtracting the ∆G of individual reactions in the methanogenesis pathway from the net reaction. ∆G for most steps were available from the literature [32–37]. For methylene-tetrahydromethanopterin reduction with F_420_H_2_, the ∆G was estimated by adding the values for F_420_H_2_ reduction of H^+^ [38] and H_2_-driven reduction of methylene-tetrahydromethanopterin [39]. Using these ∆G values, reduction potentials of −143, −385, and −520 mV for coenzyme M/B disulfide, F_420_ and ferredoxin respectively [38, 40, 41], ATP hydrolysis ∆G of −60 kJ mol^−1^, transmembrane H^+^ and Na^+^ transport ∆G of −20 kJ mol^−1^, limit (quasi-equilibrium) metabolite concentrations were calculated as described by González-Cabaleiro et al. [42].

### Resting cell experiment with *M. shengliensis* ZC-1

*M. shengliensis* ZC-1 cells grown in 50 mL medium (see above) with 10 mM TMB as substrate were harvested under anoxic conditions in the exponential phase and washed with stabilization buffer (2 mM KH_2_PO_4_/K_2_HPO_4_, 2 mM MgSO_4_, 400 mM NaCl, 200 mM sucrose, pH 6.8). The cell pellets were resuspended in 40 ml stabilization buffer (see above) and transferred into 120 ml anaerobic glass bottles (OD 0.1). The cultures were incubated for 30 min at 65 °C. Afterwards, TMB was added to a final concentration of 10 mM and the cultures were incubated for 6 h at 65 °C. The CH_4_ and CO_2_ gas produced by the cultures was analyzed every hour by injecting 50 μL headspace volume with a gas-tight glass syringe (Hamilton, Reno, NE) into an Agilent 6890 series gas chromatograph coupled to a mass spectrometer (GC-MS) (Agilent, Santa Clara, CA) equipped with a Porapak Q column heated at 80 °C. For calculating the percentage of CH_4_ and CO_2_ in the culture headspace a calibration curve was generated by injecting different volumes of calibration gas (Linde Gas Benelux) that contained 1% CO_2_ and 1% CH_4_ into the GC-MS. The CO_2_ values (in %) were corrected for the CO_2_ in the medium in form of HCO_3_^−^ by measuring the headspace CO_2_ in 40 ml buffer before and after acidification with HCl. The experiment was performed in triplicate.

### [^13^C] labeled bicarbonate experiment

*M. shengliensis* ZC-1 cells were incubated in 50 mL medium (see above; instead of [^12^C] bicarbonate, [^13^C] bicarbonate was used) with 10 mM TMB or 75 mM MeOH as substrate at 65 °C in quadruplicates. This experiment has to be interpreted in a qualitative and not a quantitative way as not all of the CO_2_ present in the cultures is [^13^C] CO_2_. The medium was sparged with N_2_:CO_2_ (80:20) and the CO_2_ in the gas was not [^13^C] labeled. The carbonate buffering system is required for growth of those cultures. The [^12^C]- and [^13^C]-CH_4_ and CO_2_ gas produced by the cultures was analyzed every day for a period of 7 days by injecting 50 μL headspace volume into a GC-MS (see above). The ratio of [^12^C] and [^13^C] CH_4_ was calculated for all cultures to different time points (day 4 to 7). The 1% [^13^C]-CH_4_ naturally present in CH_4_ was subtracted from the [^13^C]-CH_4_ values.

## Results and discussion

### Genomic analysis

Anaerobic degradation of methyl compounds in both *Archaea* and *Bacteria* begins with the transfer of the methyl group to a physiological C_1_ carrier. In both systems, a substrate-specific methyltransferase (MT1; Eq. 1) transfers the methyl group to a corrinoid protein (CP) and another methyltransferase (MT2 Eq. 2) performs a subsequent transfer to a physiological C_1_ carrier—coenzyme M (CoM) for *Archaea* and tetrahydrofolate (H_4_F) for *Bacteria* [14, 43]. Both require two methyltransferases, one CP, and an activating enzyme to recycle adventitiously oxidized CPs [44, 45].

1 $${\mathrm{CH}}_3--{\mathrm{X}} + {\mathrm{Co}}( {\mathrm{I}})--{\mathrm{CP}} + {\mathrm{H}}^ + \to {\mathrm{CH}}_3--{\mathrm{Co}}({{\mathrm{III}}})--{\mathrm{CP}} + {\mathrm{H}}--{\mathrm{X}}$$

2 $${\mathrm{CH}}_3--{\mathrm{Co}}({{\mathrm{III}}})--{\mathrm{CP}} + {\mathrm{Y}}--{\mathrm{H}} \to {\mathrm{CH}}_3--{\mathrm{Y}} + {\mathrm{Co}}( {\mathrm{I}})--{\mathrm{CP}} + {\mathrm{H}}^ +$$

Y = CoM or H_4_F

**Fig. 1:**
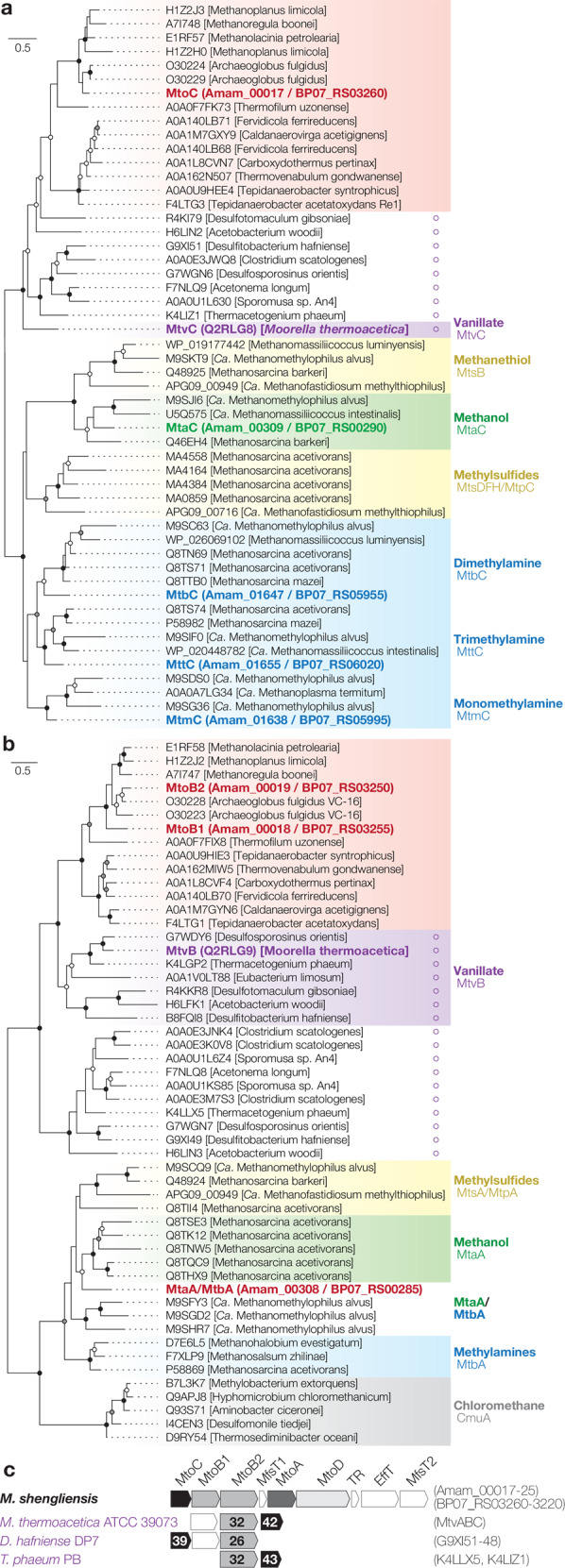
*M. shengliensis* AmaM and ZC-1 corrinoid protein and methyltransferase phylogeny. **a** A phylogenetic tree of AmaM methyltransferase corrinoid proteins (red and bolded) and homologs were generated through sequenced alignment via MAFFT v7.394 and tree calculation via RAxML-NG v0.5.1b. The homologs include those specific to vanillate (MtvC; purple), MeOH (e.g., MtaC; green), methylated thiols (e.g., MtsB; yellow), and methylamines (e.g., MtmC; blue). For methyltransferase corrinoid proteins fused with their partner methyltransferase, only the cobalamin-binding region was extracted for this alignment. In addition, a novel cluster of bacterial methyltransferases is shown, including those from ArOCH_3_-metabolizing anaerobes (indicated with purple circles). Bootstrap values are shown for 200 iterations (>90% black, >70% gray, >50% white). **b** Phylogenetic tree of MtaA/CmuA family (TIGR01463, cd03307, and IPR006360) and MtvB-related methyltransferases, including those from *M. shengliensis* (red and bolded) and *M. thermoacetica* (purple and bolded). MT2 for MeOH (e.g., MtaA; green), methylamine (e.g., MtbA; blue), and MeOH/methylamine bifunctionally; bifunctional MT1/MT2 for methylated thiols (e.g., MtsA; yellow); and MT1 for chloromethane (gray) are shown. Methyltransferases affiliated with ArOCH_3_-metabolizing anaerobes (purple circles) form a novel cluster. **c** The operon encoding the novel corrinoid protein (MtoC) with methyltransferases (MtoB1, MtoB2, and MtoA) and corrinoid protein activase (MtoD) along with potential aromatic compound transporters (MfsT MFS transporter, EffT Efflux transporter) and a transcriptional regulator (TR). Operons identified in bacterial ArOCH_3_ metabolizers are also shown with amino acid sequence percent identity with MtoC and MtoB2.

*M. shengliensis* can metabolize MeOH and mono-, di-, and tri-methylamines and encodes the necessary substrate-specific CPs, MT1s, and MT2s (Fig. 1a, b; Supplementary Table S1). However, with help of an extensive genome analysis we identified an operon in the *M. shengliensis* AmaM and ZC-1 genomes encoding a putative methyltransferase complex of unknown specificity that has previously been overlooked. This operon includes a CP (Amam_00017; BP07_RS03260), three methyltransferases (Amam_00018, Amam_00019, and Amam_00021; BP07_03250, BP07_03255, and BP07_RS03240), and a corrinoid activator protein (Amam_00022; BP07_RS03235) distantly related to known methanogen methyltransferase components (Fig. 1a–c, and Supplementary Fig. S1).

Although an archaeal *O*-demethylase/methyltransferase system for methoxylated aromatic compounds has not been described previously, some genes identified in this study and mentioned above show homology with counterparts in the bacterial Mtv *O*-demethylation system present in the homoacetogenic bacterium *Moorella thermoacetica* (Pierce et al. [60]; Fig. 1a, b; Supplementary Table S1). Amam_00017 (BP07_RS03260) and Amam_00018/19 (BP07_RS03255/50) are closely related to the CP (MtvC) and vanillate-specific MT1 (MtvB) of the three-component *Moorella thermoacetica* vanillate *O*-demethylase system MtvABC [46], indicating involvement of the operon in ArOCH_3_ demethylation. We also found Amam_00017, 18, and 19 homologs in the genomes of other ArOCH_3_-catabolizing bacterial anaerobes whose methyltransferases have yet to be identified (Fig. 1c) [47–51]. Based on phylogenetic comparison of the archaeal and bacterial systems, Archaea likely acquired the *O*-demethylase (MtvB) and corresponding CP (MtvC) for methoxylated aromatic compound metabolism through horizontal gene transfer from Bacteria (Fig. 1a, b). The genes putatively involved in methoxydotrophic growth are also present in other archaea like *Archaeoglobus fulgidus* and the hydrogenotrophic methanogens *Methanolacinia petrolearia* and *Methanothermobacter tenebrarum* (Fig. 1a, b), indicating that the trait for methoxydotrophic growth might be more prevalent among archaea than previously thought.

As the above methyltransferases and CP are cytosolic, *M. shengliensis* requires transporters for the uptake of methoxylated aromatic compounds. Although specific transporters for aromatic compounds have not been found for methanogens, previous studies have characterized several bacterial aromatic acid:H^+^ symporters belonging to the major facilitator superfamily (MFS) [52]. This includes PcaK from *Pseudomonas putida* [53], TfdK from *Ralstonia eutropha* [54], BenK, VanK, PcaK, and MucK from *Acinetobacter* sp. ADP1 [55–57] and MhpT from *Escherichia coli* [58]. We also identified genes encoding MFS transporters adjacent to the aforementioned methyltransferases (Fig. 1c and Supplementary Table S1) and suspect that they drive aromatic compound transport for *M. shengliensis*.

### Novel demethoxylation pathway involves methyl transfer to tetrahydromethanopterin

**Fig. 2:**
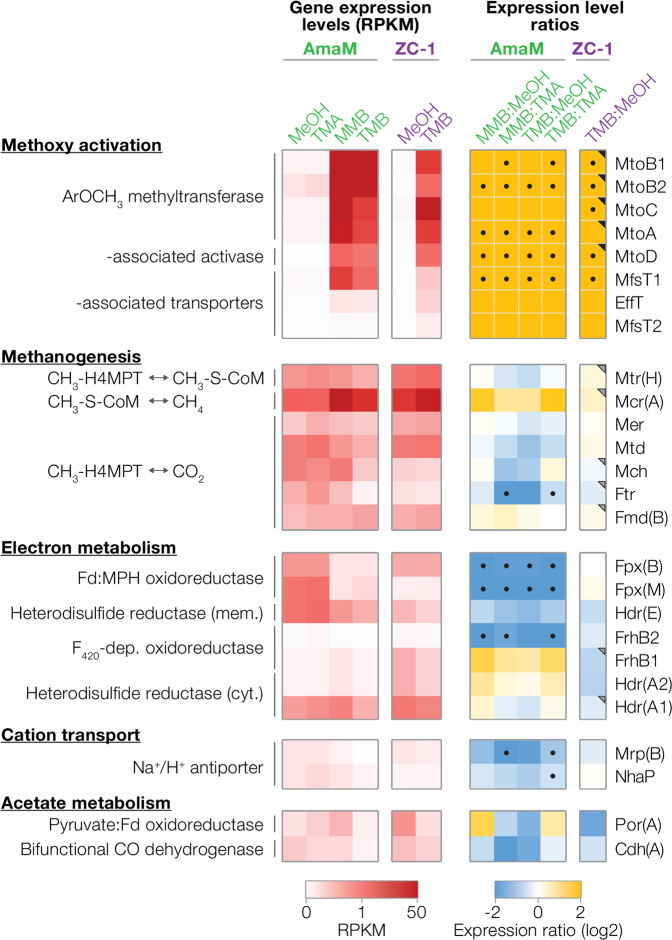
Comparison of gene expression during growth on methylated and methoxylated substrates. (left) Gene expression of the novel AmaM/ZC-1 corrinoid protein and methyltransferase operon, methanogenesis pathways, and electron transduction (see Supplementary Table S1 for abbreviations). RPKM (reads per kilobase transcript per million mapped reads) values are normalized to the average ribosomal protein RPKM under methanogenesis from MeOH, trimethylamine (TMA), 2-methoxybenzoate (MB), and trimethyoxybenzoate (TMB). (right) The ratios of gene expression between ArOCH_3_- and methylated compound-fed conditions are shown (*p* value < 0.05 marked with dot). For ZC-1, dots are shown if at least two TMB-grown cultures show significantly different RNA expression levels (*p* value < 0.05) from the MeOH-grown cultures (see Supplementary Table S1). Similarly, triangles are shown if significant differences in protein expression levels were observed (*p* value < 0.05). For entries spanning multiple genes, expression levels of specific subunits are shown as indicated on the right-hand side.

To verify involvement of the aforementioned gene cluster in ArOCH_3_ metabolism, we compared AmaM transcriptomes during methanogenesis from ArOCH_3_ (i.e., *p*-methoxy-benzoate [MB] and 3,4,5-trimethoxybenzoate [TMB]) and methyl compounds (i.e., MeOH and trimethylamine) as well as the ZC-1 transcriptomes and proteomes of ArOCH_3_ (i.e., TMB)—and cells grown on methyl compounds (i.e., MeOH). For AmaM, the MtvB-related methyltransferase MtoB2, another methyltransferase designated MtoA, reductive activase MtoD, and an MFS transporter MfsT1 were consistently strongly upregulated during growth on methoxylated aromatic compounds (*p* value <  0.05; Fig. 2 and Supplementary Tables S1 and S2). Similarly, ZC-1 upregulated MtoB1, MtoB2, CP MtoC, reductive activase MtoD, and MfsT1 in the transcriptomes and or proteomes (*p* value < 0.05; Fig. 2 and Supplementary Table S1). The novel *M. shengliensis* methyltransferase genes displayed one of the highest increases in expression among all genes (Fig. 2 and Supplementary Table S1), up to 90-fold. We propose that Amam_00017~22/BP07_RS03235~60 collectively function as a novel ArOCH_3_-specific *O*-demethylase/methyltransferase system, tentatively termed the Mto system based on the nomenclature used by Sauer and Thauer for methanogenic methyltransferases [59], and the adjacent transporters as ArOCH_3_ uptake or byproduct aromatic compound efflux proteins. We further propose that Amam_00018/BP07_RS03255 and Amam_00019/BP07_RS03250 function as ArOCH_3_-specific MT1 (MtoB1 and MtoB2 respectively) and Amam_00017/BP07_RS03260 as the corresponding methyl-carrying CP (MtoC), based on the aforementioned similarity with *Moorella thermoacetica* MtvB and MtvC (Fig. 1). Together, MtoB(1/2) and MtoC likely accomplish the first step in ArOCH_3_ *O*-demethylation (Eq. 3).

3 $${\mathrm{ArOCH}}_3 + {\mathrm{Co}}({{\mathrm{I}}}) + {\mathrm{H}}^ + \to {\mathrm{CH}}_3-- {\mathrm{Co}}( {{\mathrm{III}}})--{\mathrm{MtoC}} + {\mathrm{ArOH}}$$

As described before, *M. shengliensis* can use a broad range of different methoxylated aromatics for growth [6]. The *O*-demethylase proteins MtoB1 (Amam_00018/BP07_RS03255; 48 kDa) and MtoB2 (Amam_00019/BP07_RS03250; 47 kDa) have a sequence similarity of 57% to each other (NCBI BLASTp). This dissimilarity might hint towards different substrate affinities of the two proteins. In the first step of methoxydotrophic methanogenesis, through *O*-demethylation via the MtoB proteins, the methyl group is most likely transferred to the cobalt containing CP MtoC (22 kDa; N-terminal Coenzyme B_12_ binding site [Prosite: https://prosite.expasy.org]). MtoD (Amam_00019/BP07_RS03235; 68 kDa) is predicted to perform activation of the CP, a process necessary for catalytic activity of the CP in both acetogens and methanogens. This corrinoid activation protein MtoD harbors an N-terminal 2Fe-2S binding site (Prosite: https://prosite.expasy.org), a feature more similar to those of acetogens than methanogens (two C-terminal 4Fe-4S clusters) [28].

**Fig. 3:**
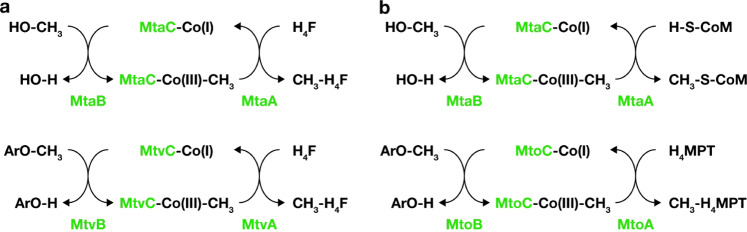
Demethylation and demethoxylation pathways in acetogenic bacteria and methanogenic archaea. **a** Demethylation and demethoxylation pathways as described for the acetogenic bacterium *Moorella thermoacetica*, modified from Pierce et al. [60]. **b** Demethylation and tentative demethoxylation pathways in methanogenic archaea. Co(I/III): oxidation state of the cobalamin carried by the cobalamin binding protein MtoC, H_4_MPT: tetrahydromethanopterin.

The next step is methyl transfer from CH_3_-MtoC to a physiological C_1_ carrier by a methyl transferase (MT2). In methylotrophic methanogens, the methyl group is transferred from the CP to CoM via the methyl transferase MtaA when grown on methanol (Fig. 3). In the acetogen *Moorella thermoacetica* the methyl transferase MtvA transports the methyl group from the CP to H_4_F [46]. *M. shengliensis* does not encode an *mtvA*-like gene and *mtaA* is neither upregulated under growth on methoxylated compounds nor part of the identified methoxydotrophy gene cluster. Instead, an *mtrH*-like gene (Amam_00021/BP07_RS03240) is part of the aforementioned operon and is highly upregulated under methoxydotrophic growth in *M. shengliensis*. This gene is not homologous to any known MT2 and rather relates to methyltransferase family PF02007: methyl-tetrahydromethanopterin (H_4_MPT):CoM methyltransferase (Mtr) subunit H (MtrH; 41% peptide similarity to that of *Methanosarcina barkeri*) (Supplementary Fig. S1). Although MtrH (subgroup I) is part of the membrane-bound Mtr complex found in methanogens, the identified *M. shengliensis* MtrH homolog Amam_00021/BP07_RS03240 relates more to MtrH-related proteins (e.g., subgroup III) that do not form such a complex and are found in non-methanogenic archaea (e.g., *Archaeoglobus fulgidus*) and methylotrophic bacteria *Desulfitobacterium hafniense* or *Acetobacterium woodii* [61] (i.e., organisms that neither synthesize nor utilize CoM). MtrH in *Desulfitobacterium hafniense* has been described as a methylcorrinoid:tetrahydrofolate methyltransferase [62]. As Amam_00021/BP07_RS03240 is upregulated together with the neighboring MtoC, MtoB1, and MtoB2 during ArOCH_3_ metabolism, we hypothesize that the gene product serves as an CH_3_-(CoIII)-MtoC:H_4_MPT methyltransferase (Eq. 4), tentatively named MtoA. Together MtoAB(1/2)C might catalyze complete methyl transfer from ArOCH_3_ to H_4_MPT (Eq. 5) and MtoD is a corresponding corrinoid activation protein required for sustained methyltransferase activity (Eq. 6):

4 $${\mathrm{CH}}_3--{\mathrm{Co}}({{\mathrm{III}}})--{\mathrm{MtoC}} \, +\, 	 {\mathrm{H}}_4{\mathrm{MPT}}\to {\mathrm{CH}}_3--{\mathrm{H}}_4{\mathrm{MPT}} \\ +\, 	 {\mathrm{Co}}( {\mathrm{I}})--{\mathrm{MtoC}}$$

5 $${\mathrm{ArOCH}}_3 + {\mathrm{H}}_4{\mathrm{MPT}} \to {\mathrm{ArOH}} + {\mathrm{CH}}_3--{\mathrm{H}}_4{\mathrm{MPT}}$$

6 $${\mathrm{Co}}( {{\mathrm{II}}})--{\mathrm{MtoC}} \, +\, 	 {\mathrm{ATP}} + {\mathrm{H}}_2{\mathrm{O}} + {\mathrm{e}}^ - \to {\mathrm{Co}}( {\mathrm{I}})--{\mathrm{MtoC}} \\ +\, 	 {\mathrm{ADP}} + {\mathrm{P}}_{\mathrm{i}}$$

**Fig. 4:**
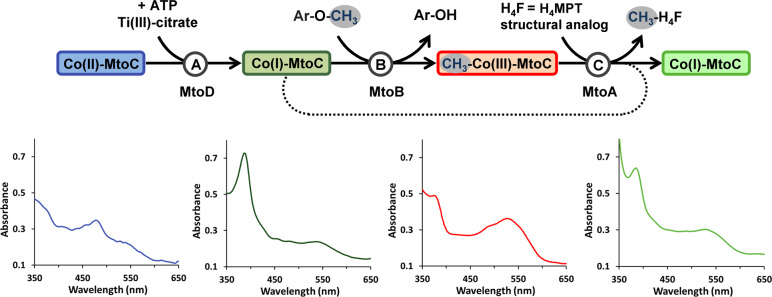
*O*-demethylation and methyl transfer conducted by Mto proteins. Reaction A: MtoD, ATP and titanium (III) citrate are required for activation of MtoC from the Co(II) state (blue) to the active Co(I) state (dark green). Reaction B: MtoB transfers the methyl group of the methoxy compound (Ar-O-CH_3_) to Co(I)-MtoC resulting in methylated Co(III)-MtoC (red). The MtoB activities with 2-methoxybenzoate (MB) and TMB are shown in Fig. S2A. With methanol or trimethylamine as substrate no activity could be observed. Conversion of TMB to 3-OH-4,5-dimethoxybenzoate was confirmed by HPLC (Fig. S3). Reaction C: For measuring MtoA activity, the H_4_MPT structural analog H_4_F was used. We got strong evidence that MtoA transfers the methyl group from methylated Co(III)-MtoC (red) to H_4_F thereby producing Co(I)-MtoC (light green). The activity is shown in Fig. S2B. In *M. shengliensis* H_4_MPT and not H_4_F is most likely the methyl group acceptor as *M. shengliensis* does not have the genomic capacity to synthesize H_4_F. Also, the methyl-transfer reaction is not occurring if CoM is used instead of H_4_F (Fig. S2B). All bottom panels correspond to UV/visible spectra measured after each reaction reflecting the different states of the cobalamin carried by MtoC.

To verify the function of the Mto proteins from *M. shengliensis* in *O*-demethylation and methyl transfer, we purified the Mto proteins and analyzed them by UV–vis spectroscopy and enzyme activity assays (Fig. 4 and Supplementary Fig. S2).

MtoC exhibits different UV–vis spectroscopic features depending on the oxidation state of its cobalamin cofactor. In the inactive Co(II) state, the UV–vis spectrum shows a peak at around 480 nm (Fig. 4, before reaction A). The corrinoid activator MtoD can reactivate the Co(II) state of MtoC by reducing the cobalamin to the active Co(I) state with the use of ATP and titanium (III) citrate (Fig. 4, reaction A). The active Co(I) state exhibits a peak at around 390 nm (gamma band). When MtoB and the methoxylated aromatic compound are added to the enzyme assay mixture the methyl group is transferred to the cobalamin (Fig. 4, reaction B), as also shown by HPLC (Supplementary Fig. S3). The formation of methyl-Co(III) provokes the disappearance of the peak at 390 nm and the appearance of a new peak at 520 nm. The demethylation of MtoC by MtoA was observed when tetrahydrofolate (H_4_F), a C1-carrier analogous to H_4_MPT, was added (Fig. 4, reaction C). This reaction can be followed by the decrease of absorbance at 520 nm and the increase of absorbance at 390 nm, which is explained by a switch back to the Co(I) state. As H_4_F instead of the native methyl acceptor H_4_MPT is used in the assay no specific activity value for MtoA could be accurately determined. By HPLC analysis of the methoxy compounds and their hydroxylated derivatives we observed that roughly 2.2% of the methoxy compound is converted (i.e., about 51 µM of the initial 2.3 mM TMB; Supplementary Fig. S3), which agrees with the concentration of the methyl-acceptor MtoC in the assay mixture (~55 µM). The MtoB activity with 2-methoxybenzoate (MB) was found to be 0.87 ± 0.04 µmol Co(III) formed per min and per mg of MtoB and with TMB 0.76 ± 0.04 µmol of Co(III) formed per min and per mg of MtoB (see also Supplementary Fig. S2A). The specific activity values of the *O*-demethylase of *Acetobacterium dehalogenans* measured with vanillate and isovanillate are 0.43 and 0.65 µmol Co(III) formed per min and per mg MT1 respectively, for example [28].

With those experiments we showed that the *O*-demethylation and methyl transfer reaction are indeed catalyzed by the Mto proteins and that this system works in a similar way as shown for methoxydotrophic bacteria such as *Moorella thermoacetica* [46] or *A. dehalogenans* [28]. We could identify MtoB (WP_042685515.1) as the *O*-demethylase catalysing the methyl transfer from the methoxy compound to Co(I)-MtoC. After accepting the methyl group from MtoB, MtoC could not be demethylated by MtoA in the presence of HS-CoM, the conventional methyl-acceptor for methylotrophic methanogenenesis. On the other hand, MtoC demethylation by MtoA could be observed when the H_4_MPT structural analog H_4_F was present. Given that *M. shengliensis* can only synthesize H_4_MPT and not H_4_F (e.g., absence of bacterial dihydrofolate reductase), this gives us strong evidence that H_4_MPT, rather than HS-CoM, should accept the methyl group from CH_3_-Co(III)-MtoC in *M. shengliensis*.

**Fig. 5:**
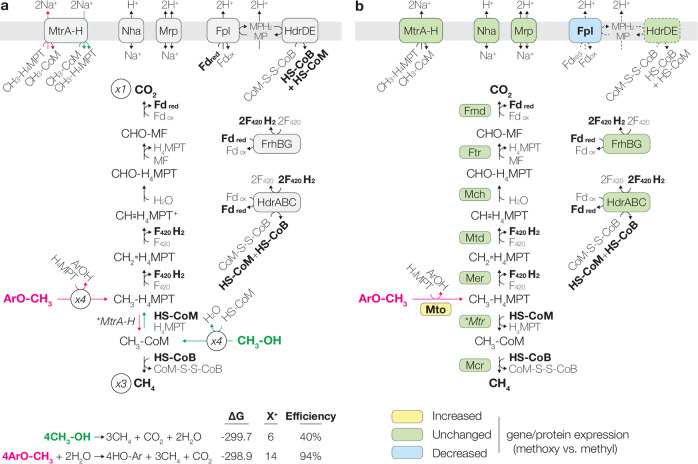
Comparison of CH_3_-CoM- and hypothetical CH_3_-H_4_MPT-disproportionating methanogenesis based on (a) energetics and (b) expression. **a** Reactions and reaction directions unique to MeOH (green) or 2-methoxybenzoate (pink) decomposition are shown. Below are the estimated Gibbs free energy (∆G) and the predicted energy yield (in terms of H^+^/Na^+^ extruded across the membrane, assuming the typical scheme of methylotrophic methanogenesis is followed) and thermodynamic efficiency of the shown methanogenesis pathways. ∆G was calculated assuming 60 °C, pH 7, 0.2 atm CO_2_, 0.2 atm CH_4_, 1 mM NH_4_^+^, and 10 mM for all other compounds. **b** Comparison of gene/protein expression of *M. shengliensis* grown on methoxylated aromatic compounds and methylated compounds. Yellow: genes/proteins for which both strains showed significantly increased expression during methoxydotrophic methanogenesis. Green: genes/proteins for which either (i) expression levels were not significantly different for both strains or (ii) consistent trends were not observed in both strains. Blue: genes/proteins for which both strains showed significantly decreased expression during methoxydotrophic methanogenesis. Arrows involving Fpl and HdrDE are dotted as Fpl was downregulated during methoxydotrophic methanogenesis and, with decreased Fpl activity, HdrDE’s activity would consequently decrease as well. H_4_MPT tetrahydromethanopterin, MF methanofuran.

Such a H_4_MPT-dependent methyl transfer would be the first of its kind though, in some aspects, comparable to other pterin-dependent methyl activation pathways—H_4_MPT/H_4_F-dependent acetyl-CoA decarbonylation and H_4_F-dependent acetogenic methyl transfer pathway [63]. If the *Methermicoccus* methyltransferase system is indeed dependent on the archaeal equivalent of H_4_F, this may be because the archaeal ability to degrade methoxylated aromatic compounds likely originated in C1-metabolizing *Firmicutes*, based on the topology of the MtoB and MtoC phylogenetic trees (Fig. 1a, b). The proposed transfer of the ArOCH_3_-derived methyl group to H_4_MPT rather than CoM would significantly influence the energetics of methanogenesis. Based on thermodynamic calculations we suggest the following hypotheses regarding the energy metabolism of methoxydotrophic methanogens: typical methylotrophic methanogenesis disproportionates CH_3_-S-CoM to ¼ CO_2_ and ¾ CH_4_. In this pathway (4CH_3_X + 2H_2_O → CO_2_ + 3CH_4_ + 4HX), CH_3_-S-CoM oxidation to CO_2_ requires an energy input (~2Na^+^ transported in for transferring the methyl group from CoM to H_4_MPT; Fig. 5) but electron transfer from this oxidation to reduction of CH_3_-S-CoM to CH_4_ allows energy recovery (~8H^+^ transported out, assuming all F_420_H_2_ is re-oxidized via Fpo-related Fd:methanophenazine (Mp) oxidoreductase (Fpl); Fig. 5). Assuming each H^+^/Na^+^ transported across the membrane stores 20 kJ per mol, this yields a net energy gain of 120 kJ per four mol methyl substrate. If methoxydotrophic methanogenesis follows an analogous pathway with an entry point at CH_3_-H_4_MPT (CH_3_-H_4_MPT disproportionation to ¼ CO_2_ and ¾ CH_4_), oxidation of CH_3_-H_4_MPT to CO_2_ would not incur any energetic cost, while reduction of CH_3_-H_4_MPT to CH_4_ would generate energy (~6Na^+^ transported out; Fig. 5). Combined with the energy gain from electron transfer (~8H^+^ transported out), such metabolism would yield a net energy gain of 280 kJ per four mol methoxylated substrate. Based on such energetics, *M. shengliensis* methylotrophic and methoxydotrophic methanogenesis would, in theory, respectively reach roughly 40% and 94% thermodynamic efficiency (e.g., 40.0, 41.2, and 93.7% for MeOH [−299.7 kJ], monomethylamine [−291.0 kJ], and 2-methoxybenzoate [−298.9 kJ] correspondingly at 60 °C, pH 7, 0.2 atm CO_2_, 0.2 atm CH_4_, 1 mM NH_4_^+^, and 10 mM for all other compounds; see also Supplementary Table S3). However, most anaerobes work at efficiencies around 25–50% and efficiencies above 80% are highly improbable [64], suggesting that methoxydotrophic methanogenesis through such a pathway would be impossible. To operate at an energetic efficiency that organisms can physicochemically achieve, methoxydotrophic methanogenesis most likely takes an alternative route that recovers a lesser amount of energy. As an analogous phenomenon of trading off energy yield for thermodynamic driving force, one can look at glycolysis—compared to the Embden-Meyerhof-Parnas pathway, the Entner-Douduroff pathway sacrifices half of the ATP yield partly to minimize thermodynamic bottlenecks and prioritize thermodynamic feasibility [65, 66]

Supporting the possibility of an alternative route (i.e., not simple disproportionation to CO_2_ and CH_4_), we obtained evidence that methylotrophic and methoxydotrophic methanogenesis behave differently metabolically—while nearly all CH_4_ (96.4%) produced from strain AmaM methylotrophic methanogenesis originated from the methylated substrate (as was also observed for *Methanosarcina barkeri* [98-99% CH_4_ from methanol; [67]), CH_4_ from strain AmaM methoxydotrophic methanogenesis originated from both the methoxylated substrate (2/3) and CO_2_ (1/3) [6]. We also compared the growth of strain ZC-1 on TMB and MeOH and, in agreement, found that the former consumes more CO_2_ for methanogenesis: in a qualitative experiment with [^13^C] bicarbonate we found that ZC-1 cells grown on TMB produced roughly 10 times more [^13^C]-CH_4_ from [^13^C]-bicarbonate-derived CO_2_ than those grown on MeOH. Thus, both strains seem to display the same atypical behavior when degrading methoxylated compounds. Given that both strains lack genes for any alternative C_1_ metabolism (e.g., aerobe-like aldehyde-based or anaerobic bacterial H_4_F-based metabolism), H_4_MPT-dependent C_1_ metabolism is presumably responsible for running both CO_2_ and CH_4_ generation from ArOCH_3_ as well as CH_4_ generation from CO_2_. In search of a metabolic route that provides a rationale for this anomalous behavior and thermodynamic efficiency, we further compare the gene expression of *M. shengliensis* when degrading methylated compounds and ArOCH_3_ to gain insight into how the pathways may differ regarding electron transport and energy recovery.

During methylotrophic growth, AmaM and ZC-1 express the corresponding methyltransferase system and the complete methanogenesis pathway (i.e., CH_3_-S-CoM disproportionation to CO_2_ and CH_4_). To transfer electrons from the oxidative to reductive pathway, the two strains express two putative ferredoxin (Fd)-dependent F_420_:CoB-S-S-CoM oxidoreductases (HdrA1B1C1 or FrhBG-HdrA2B2C2; Eq. 7) [40], a putative Fpo-related Fd:methanophenazine (Mp) oxidoreductase (FplABCDHIJKLMNO; Eq. 8; see Supplementary Table S1) [68, 69], and a Mp-oxidizing membrane-bound heterodisulfide reductase (HdrDE; Eq. 9).

7 $$2{\mathrm{F}}_{420}{\mathrm{H}}_2 \, +\, 	 {\mathrm{Fd}}_{{\mathrm{ox}}} + {\mathrm{CoM}}--{\mathrm{S}}--{\mathrm{S}}-- {\mathrm{CoB}}\\ 	 \to 2{\mathrm{F}}_{420} + {\mathrm{Fd}}_{{\mathrm{red}}}+ {\mathrm{HS}}--{\mathrm{CoM}} + {\mathrm{HS}}--{\mathrm{CoB}}$$

8 $${\mathrm{Fd}}_{{\mathrm{red}}} + {\mathrm{Mp}} + 4{\mathrm{H}}_{{\mathrm{in}}}^ + \to {\mathrm{Fd}}_{{\mathrm{ox}}} + {\mathrm{MpH}}_2 + 2{\mathrm{H}}_{{\mathrm{out}}}^ +$$

9 $${\mathrm{MpH}}_2 	+ {\mathrm{CoM}}--{\mathrm{S}}--{\mathrm{S}}--{\mathrm{CoB}}+2{\mathrm{H}}^+\\ 	\to {\mathrm{Mp}} + {\mathrm{HS}}--{\mathrm{CoM}} + {\mathrm{HS}}--{\mathrm{CoB}}+2{\mathrm{H}}_{{\mathrm{out}}}^ +$$

Combined together, these complexes can facilitate complete electron transfer for CH_3_-S-CoM disproportionation (Eq. 7 + 2x Eq. 8 + 2x Eq. 9; Fig. 5). However, during methoxydotrophic growth, we observe significant decreases in the expression of Fpl compared to methylotrophy (Supplementary Table S1): 3.8–10.1 fold decrease for FplBIMN in AmaM transcriptomes (*p* < 0.043) and 3.2 fold decrease for FplD in ZC-1 proteomes (*p* = 0.0027). Although decreases in all Fpl subunits were not observed, the downregulated subunits play critical roles in the activity of the Fpl complex – FplBI and FplD are predicted to mediate Fd_red_ oxidation [68] and interaction with the transmembrane subunits, respectively. Thus, both AmaM and ZC-1 might decrease electron transfer via Fpl and then would have to redirect intracellular electron flow through an alternative pathway. Interestingly, Fpl is central to energy generation from electron transfer (Eqs. 7 and 8), suggesting that *M. shengliensis* switches to an energy acquisition scheme distinct from that of methylotrophic methanogenesis. In other words, while methylotrophic methanogenesis gains energy purely from electron transfer (F_420_H_2_ re-oxidation), methoxydotrophic methanogenesis may forgo such energy metabolism and rather gain energy from methyl transfer (CH_3_-H_4_MPT to CH_3_-S-CoM).

Based on the annotatable genes for methanogenesis and energy metabolism expressed by *M. shengliensis*, the above electron transfer/energy acquisition scheme cannot accomplish complete electron transfer from CH_3_-H_4_MPT oxidation to CH_3_-H_4_MPT reduction (Fig. S4; see Supplementary Material “Electron transfer metabolism” including Figs. S5 and S6). There is a possibility that *M. shengliensis* possesses genes that encode a novel electron transfer metabolism, but, assuming that this is not the case, ArOCH_3_ disproportionation would result in accumulation of reducing power distributed among multiple electron carriers (e.g., through activity of a ferredoxin:F_420_ oxidoreductase and HdrABC). Given that methoxydotrophic methanogenesis was observed to reduce CO_2_ to CH_4_, switching to CO_2_-reducing methanogenesis may allow cells to re-oxidize excess reducing power. Based on a thermokinetic model (see Supplementary Material; Fig. S7), cells could potentially passively alternate between oxidative (ArOCH_3_ disproportionation) and reductive (CO_2_-reducing methanogenesis) metabolism as the cells respectively approach thermodynamic and kinetic limits through accumulation or consumption of cellular reducing power. Although not found in methanogens yet, such repeated intracellularly triggered reversals in metabolism (“metabolic oscillation” or “intracellular feedback loops”) involving fluctuation of reducing power (i.e., NADH) have been observed in various organisms, including *Klebsiella sp*. (succinate or glycerol metabolism) [70, 71] and *Saccharomyces cerevisiae* (glucose) [72]. These oscillations occur on the scale of seconds to hours and concomitantly perform repeated cycles of production and consumption of metabolic end-products (e.g., CO_2_, H_2_, ethanol, or acetate) [71, 73] and intermediates (e.g., ATP) [74]. The proposed theoretical oscillation between oxidative CO_2_-/CH_4_-liberating CH_3_-H_4_MPT disproportionation and CO_2_-reducing methanogenesis is in line with the predicted need for an alternative electron transfer route (i.e., forgoing energy gain via Fpl and Hdr) and concomitant CO_2_ generation/consumption during methoxydotrophic methanogenesis, but certainly requires verification.

## Conclusion

In this study, we analysed the growth of the demethoxylating methanogen *M*. *shengliensis* on methoxylated aromatic compounds and showed that this archaeon uses a demethoxylation system (Mto) similar to those found in acetogenic bacteria. In contrast to the methylotrophic pathway of methanogenic archaea, the methyl group derived from the methoxylated compound is most likely transferred to H_4_MPT instead of CoM. In theory, such activation would thermodynamically require that methoxydotrophic methanogenesis takes an energy acquisition strategy distinct from that of methylotrophic methanogenesis. This hypothesis can be supported by the finding that, during methoxydotrophy, *M. shengliensis* downregulates genes involved in energy-generating electron transfer metabolism that is essential for methylotrophy. Clearly, methoxydotrophic methanogenesis exhibits several interesting features that differ from methylotrophic methanogenesis and requires further investigation to verify the biochemistry of methoxylated aromatic compound activation and downstream energy metabolism.

## Supplementary information

Supplementary figures

Supplementary Table 1

Supplementary Table 2

Supplementary Table 3

## Data Availability

The mass spectrometry proteomics data have been deposited to the ProteomeXchange Consortium via the PRIDE [75] partner repository with the dataset identifier PXD018934. The transcriptomics data have been deposited under GenBank SRR11935466-SRR11935483.
