# Supplementary material for: Methanogenic archaea use a bacteria-like methyltransferase system to demethoxylate aromatic compounds
Source: ISME J. 2021 Jun 18;15(12):3549–65. doi: 10.1038/s41396-021-01025-6 (PMC8630106; doi:10.1038/s41396-021-01025-6)
Supplement: Supplementary file 1 — Supplementary figures [file 41396_2021_1025_MOESM1_ESM.docx]

**SUPPLEMENTARY RESULTS**

**Growth of *M. shengliensis* ZC-1 on methoxylated aromatic compounds**

Before conducting transcriptomic and proteomic analysis, we first optimized the growth conditions of *M. shengliensis* ZC-1 on methanol as well as the methoxylated aromatic compound 3,4,5-trimethoxybenzoate (TMB). By following OD_600_ and methane production, we calculated a doubling time of 6-8 h during growth on methanol and 7-8 h when cultures were grown on TMB (Fig. S5). Overall growth on methanol and TMB is completed in about 4 days which is a considerable improvement in comparison to the 20 days that were required for growth of *M. shengliensis* on methoxylated compound previously.

**Electron transfer metabolism**

With decreased activity of Fpl (and thus HdrDE), electron transfer between CH_3_-H_4_MPT oxidation to CO_2_ and CH_3_-H_4_MPT reduction to CH_4_ cannot proceed at a 1:3 ratio. Given HdrABC (eq. 7) as the only discernable mode of intracellular electron transfer, ArOCH_3_ disproportionation to CO_2_ and CH_4_ can only incompletely proceed at a 1:1 ratio (*i.e.*, leaving excess reducing power; eq. 10).

2ArOCH_3_ + 2H_2_O → 2ArOH + CO_2_ + CH_4_ + 4H^+^ + 4e^-^ (eq. 10)

As both AmaM and ZC-1 lack the ability to reductively generate H_2_ from H^+^ (no hydrogenases), formate from CO_2_ (no formate dehydrogenases; no active site conserved in FdhA homologs), and acetate from CO_2_ (no acetate accumulation detected [1]), the most probable electron disposal route is CO_2_-reducing CH_4_ production (eq. 10).

8e^-^ + CO_2_ + 8H^+^ → CH_4_ + 2H_2_O (eq. 11)

In total, this indicates that *M. shengliensis* combines (1) oxidative ArOCH_3_ disproportionation to CO_2_ and CH_4_ (eq. 10; Fig. S4a) and (2) subsequent electron disposal through CO_2_ reduction to CH_4_ (eq. 11; Fig. S4b). Unlike methylotrophic methanogenesis in which all the generated CH_4_ originates from the methylated substrate (*e.g.*, MeOH), balancing the above reactions (2*eq. 10 + eq. 11) shows that CH_4_ generated from methoxydotrophic methanogenesis ought to originate from both the methoxylated substrate (~2/3; *i.e.*, 2 from eq. 10) and CO_2_ (~1/3; *i.e.*, 1 from eq. 11). This rationalizes our previous unusual isotope-based detection of CO_2_ reduction to CH_4_ only during methanogenesis from ArOCH_3_ by *M. shengliensis* AmaM. Moreover, the prediction that 1/3 of the generated CH_4_ is CO_2_-derived is quite consistent with our previous observation (*i.e.*, 29.6 %). We also show that resting TMB-grown *M. shengliensis* cells produce CO_2_ and CH_4_ at a 1:2 ratio (Fig. S6) rather than 1:3 expected from methylotrophy, further indicating that *M. shengliensis* methoxydotrophy (i) cannot simply disproportionate methyl groups like in methylotrophy and (ii) might accumulate reducing power (*i.e.*, the net reaction must finally reach a 1:3 ratio to balance the accumulated reducing power).

Importantly, the metabolic pathways for ArOCH_3_ oxidation to CO_2_ and CO_2_ reduction to CH_4_ overlap and operate in opposite directions, so *M. shengliensis* cannot simultaneously perform these metabolisms and must theoretically cycle between the pathways. In the following, we propose a hypothesis for this metabolic oscillation during methoxydotrophic growth of *M. shengliensis* . In theory, as ArOCH_3_ disproportionation proceeds and reducing power (RP; *e.g.*, Fd_red_ and F_420_H_2_) accumulates, the oxidative steps would decrease in thermodynamic favorability. Eventually, one of these steps would become endergonic or one of the intermediates’ concentrations would decrease to a point where the next step approaches a biologically irrelevant reaction rate. At this point, *M. shengliensis* hits a thermodynamic or kinetic wall and must switch to re-oxidation of the accumulated RP through reduction of CO_2_ to CH_4_. Initially, with the accumulated RP, CO_2_ reduction to CH_4_ is thermodynamically favorable; however, as the RP concentration decreases, the reductive steps would also decrease in thermodynamic favorability. Following the logic above, CO_2_-reducing CH_4_ generation would approach a thermodynamic or kinetic limit and force *M. shengliensis* to switch back to ArOCH_3_ disproportionation. Employing thermo-kinetic analysis based on quasi-equilibrium calculations [2], we calculated the RP concentration limits of ArOCH_3_ disproportionation and CO_2_ reduction to CH_4_ and identified the bottleneck metabolic steps. We employed three assumptions used by Gonzalez-Cabaleiro *et al*. [2]. Assuming that anaerobic microbial metabolism operates very close to thermodynamic equilibrium [3], this enables us to calculate metabolite concentrations. The total concentration of specific molecules (F_420_[H_2_], ferredoxin, XS-CoM, XS-CoB, X-H_4_MPT, and X-methanofuran) categorized as conserved moieties were presumed to remain constant [4], which allowed us to fix the total concentration of methanogenesis-related cofactors and electron carriers and simplify the above calculation. Given that very low metabolite concentrations will likely impose kinetic limitations, we also assumed that cellular metabolite concentrations below 0.1 µM will kinetically disable the reaction [5–7]. In addition, given that the concentration of methanogen cellular reducing power is known to change over the course of growth [8, 9], we made the new assumption that the excess cellular RP can accumulate or be expended (*e.g.*, cellular ratio of Fd_red_ to Fd_ox_ can increase or decrease) and that the reaction carried out by HdrABC (eq. 6) is always at equilibrium. Based on these assumptions, ∆G values and conditions described in the methods, caption of Fig. S4, and Supplementary Table S3, ArOCH_3_ disproportionation has an upper RP limit of 1.2 Fd_red_:Fd_ox_, 0.28 HS-CoM*HS-CoB:CoM-S-S-CoB, and 0.071 F_420_H_2_:F_420_ where formyl-MF oxidation to CO_2_ becomes endergonic (using 1:1 X_red_:X_ox_ at standard reduction potentials ) (Fig. S7 and Supplementary Table S3).

CO_2_ reduction to CH_4_ has a lower RP limit of 0.7 Fd_red_:Fd_ox_, 0.15 HS-CoM*HS-CoB:CoM-S-S-CoB, and 0.037 F_420_H_2_:F_420_ at which methylene-H_4_MPT and methenyl-H_4_MPT concentrations become kinetically limiting. We confirmed that ArOCH_3_ disproportionation and CO_2_ reduction to CH_4_ are respectively thermo-kinetically feasible at the lower and upper RP limits defined above. Based on our calculations, only the ∆G of formyl-methanofuran dehydrogenase flips between methoxy-disproportionation and CO_2_-reducing methanogenesis (*i.e.*, exergonic in the oxidative and reductive directions respectively) while the other reversed steps remain at equilibrium, suggesting that the thermodynamic direction of the formyl-methanofuran dehydrogenase determines the direction of the reversible arm (CH_3_-H_4_MPT ↔ CO_2_). Thus, quasi-equilibrium calculations demonstrate that *M. shengliensis* can theoretically cycle between ArOCH_3_ disproportionation and CO_2_ reduction to CH_4_ by oscillating between thermokinetic “walls”.

1. Mayumi D, Mochimaru H, Tamaki H, Yamamoto K, Yoshioka H, Suzuki Y, et al. Methane production from coal by a single methanogen. *Science* 2016; **354**: 222–225.

2. González-Cabaleiro R, Lema JM, Rodriguez J, Kleerebezem R. Linking thermodynamics and kinetics to assess pathway reversibility in anaerobic bioprocesses. *Energy Environ Sci* 2013; **6**: 3780–3789.

3. Jackson BE, McInerney MJ. Anaerobic microbial metabolism can proceed close to thermodynamic limits. *Nature* 2002; **415**: 454–456.

4. Reich JG, Sel’kov EE. Energy metabolism of the cell: a theoretical treatise. 1981. Academic Press, New York.

5. Bennett BD, Kimball EH, Gao M, Osterhout R, Van Dien SJ, Rabinowitz JD. Absolute metabolite concentrations and implied enzyme active site occupancy in Escherichia coli. *Nat Chem Biol* 2009; **5**: 593–599.

6. Bar-Even A, Noor E, Flamholz A, Buescher JM, Milo R. Hydrophobicity and charge shape cellular metabolite concentrations. *PLOS Comput Biol* 2011; **7**: 1–7.

7. Bar-Even A, Flamholz A, Noor E, Milo R. Thermodynamic constraints shape the structure of carbon fixation pathways. *Biochim Biophys Acta* 2012; **1817**: 1646–1659.

8. Heine-Dobbernack E, Schoberth SM, Sahm H. Relationship of intracellular coenzyme F_420_ content to growth and metabolic activity of *Methanobacterium bryantii* and *Methanosarcina barkeri*. *Appl Environ Microbiol* 1988; **54**: 454–459.

9. Peck MW. Changes in concentrations of coenzyme F420 analogs during batch growth of *Methanosarcina barkeri* and *Methanosarcina mazei*. *Appl Environ Microbiol* 1989; **55**: 940–945.

**SUPPLEMENTARY FIGURES**


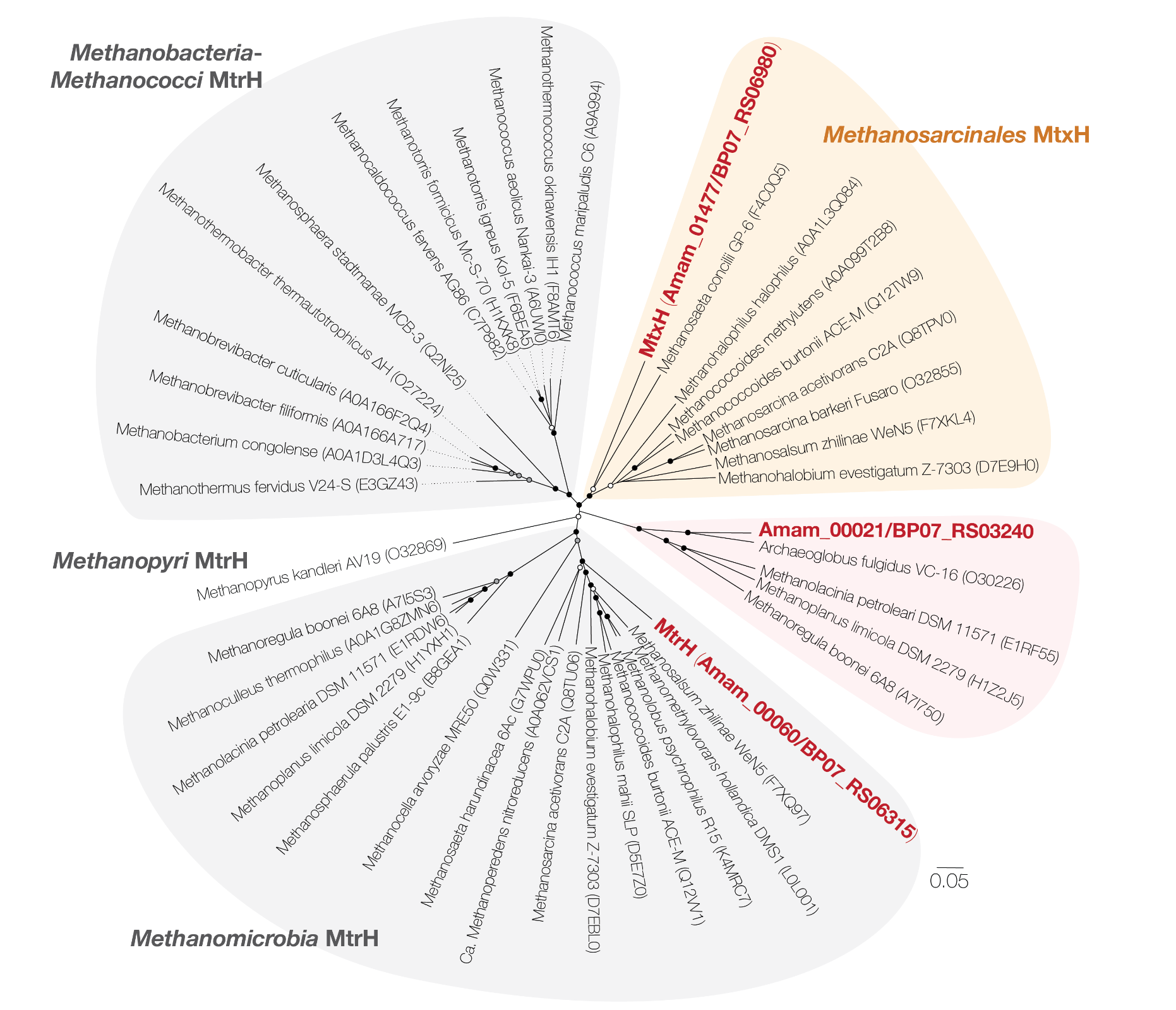


**Figure S1. Phylogenetic tree of methyl-tetrahydromethanopterin:coenzyme M methyltransferase subunit H.** MtrH homologs were collected from the *M. shengliensis* genomes and representative archaeal genomes (UniProt accession numbers). The sequences were aligned using MAFFT v7.394 and the tree was calculated using FastTree 2.1.11 with 1000 bootstrap replicates (≥90% – black circles; ≥75% – gray; ≥50% – white).


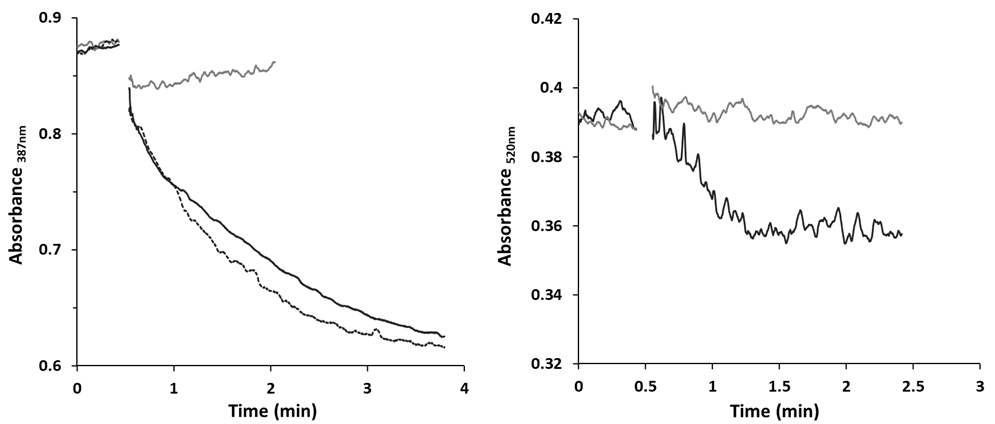


**Figure S2. Enzyme activity assay with MtoB and MtoA.** A) The *O*-demethylase MtoB transfers the methyl group of a methoxylated aromatic compound to the Co(I)-MtoC resulting in methylated Co(III)-MtoC. This reaction can be followed by a decrease in absorbance at 387 nm. With 3,4,5-trimethoxybenzoate (black dotted line) and 2-methoxybenzoate (black straight line) activity could be measured, but not with methanol as substrate (grey line). The MtoB activity with 2-methoxybenzoate (MB) was found to be 0.87 ± 0.04 µmol Co(III) formed per min and per mg of MtoB and with TMB 0.76 ± 0.04 µmol of Co(III) formed per min and per mg of MtoB. B) Methyl transfer from methylated Co(III)-MtoC to H_4_F (structural analog of H_4_MPT) results in a decrease in absorbance at 520 nm (black line). The reaction stops soon after addition of MtoA which might be due to inhibitory effects of H_4_F such as blocking the active site as it is not the native substrate. When CoM is used as methyl group acceptor (grey line) no activity could be observed.


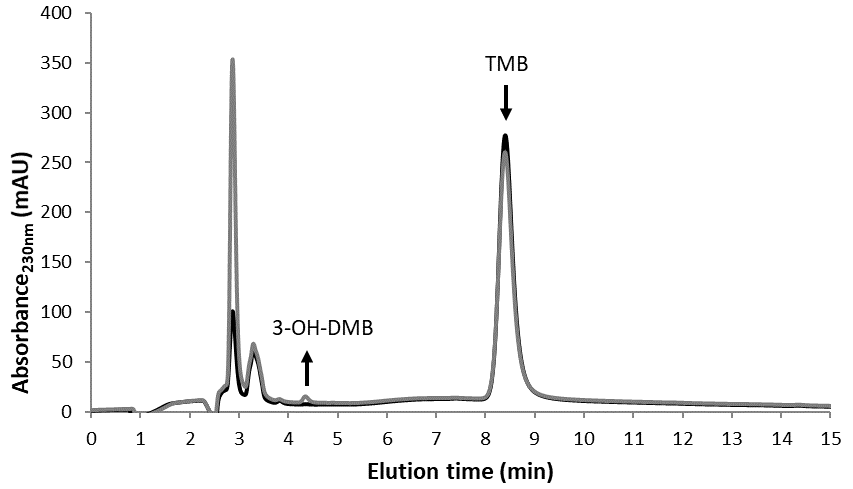


**Figure S3. HPLC analysis of samples from MtoB-dependent methoxy compound conversion measured in activity assays.** MtoB activity was determined in enzyme activity assays with 2.3 mM 2-methoxybenzoate or TMB. 50 µl sample were removed before addition of MtoB and after the activity assay for analysis of methoxy compounds by HPLC. The elution times of the standards for TMB, 3-OH-4,5-dimethoxybenzoate (3-OH-DMB) and 4-OH-3,5-dimethoxybenzoate were determined to be 8.3 min, 4.4 min and 3.9 min. 3-OH-DMB was previously found to be the main product obtained from TMB degradation in *M. shengliensis*. After addition of MtoB the amount of TMB decreases and a peak corresponding to the elution volume of 3-OH-DMB appeared. About 2.2 % of the methoxy compound was converted to the demethoxylated product after MtoB addition which matches the concentration of the methyl-acceptor MtoC in the assay (55 µM).


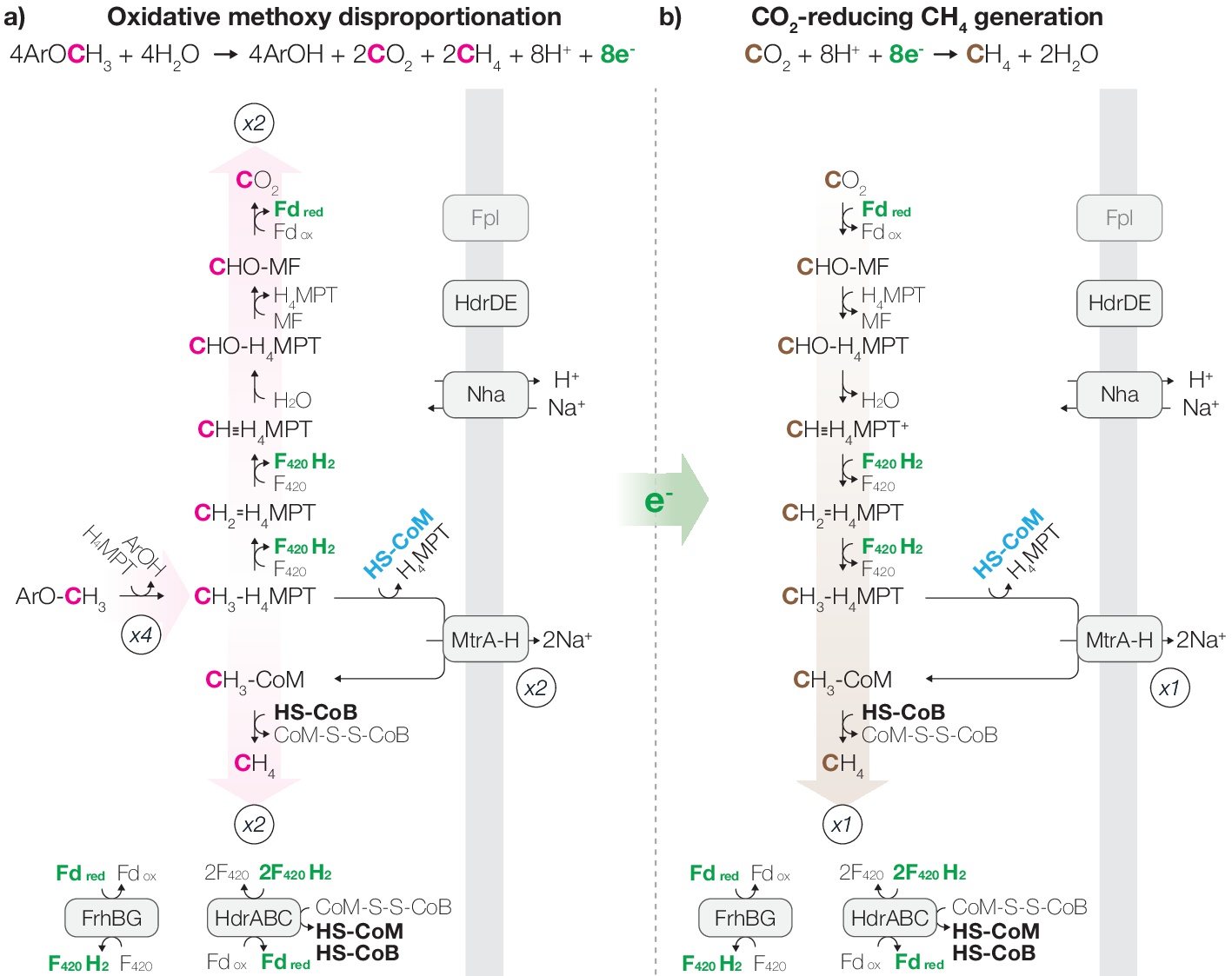


**Figure S4. Stepwise methoxydotrophic methanogenesis mediated by oxidative CH_3_-H_4_MPT disproportionation, reducing power accumulation, and CO_2_-reducing methanogenesis.** (left) Oxidative methoxy disproportionation to CO_2_ and CH_4_ at a 1:1 ratio. C_1_ molecules derived from ArOCH_3_ are marked (bolded magenta) Excess reducing power (Fd_red_ and F_420_H_2_) are highlighted (green). Steps downregulated during 2-methyoxybenzoate- and 3,4,5-trimethoxybenzoate compared to MeOH and trimethylamine are grayed out. (right) Excess reducing power from methoxy disproportionation feeds into CO_2_ reducing methanogenesis. This reducing power is theoretically distributed among Fd_red_, F_420_H_2_ based on the combined activity of HdrABC and FrhB (homologous to FpoF). C_1_ molecules originating from CO_2_ are marked (bolded cyan). The shown activity of HdrABC and FrhB are theoretical and requires further biochemistry-based verification. H_4_MPT: tetrahydromethanopterin, MF: methanofuran.


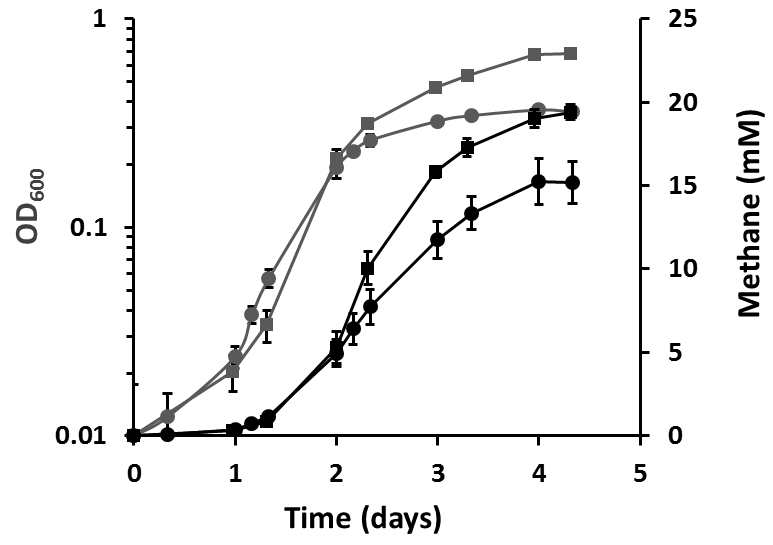


**Figure S5. Growth of *M. shengliensis* ZC-1 on TMB and methanol.** *Methermicoccus shengliensis* ZC-1 was grown either on 150 mM methanol (squares) or on 10 mM TMB (circles). Optical density at 600 nm (grey symbols) and methane production (black symbols) was measured over a period of about 4 days. The doubling time is 7.6 ± 0.4 h for growth on methanol and 8.1 ± 0.5 h for growth on TMB when calculated with OD_600_ values. Following methane production, the calculated doubling times were 6.3 ± 0.0 h for growth on methanol and 7.5 ± 0.3 h for growth on TMB.


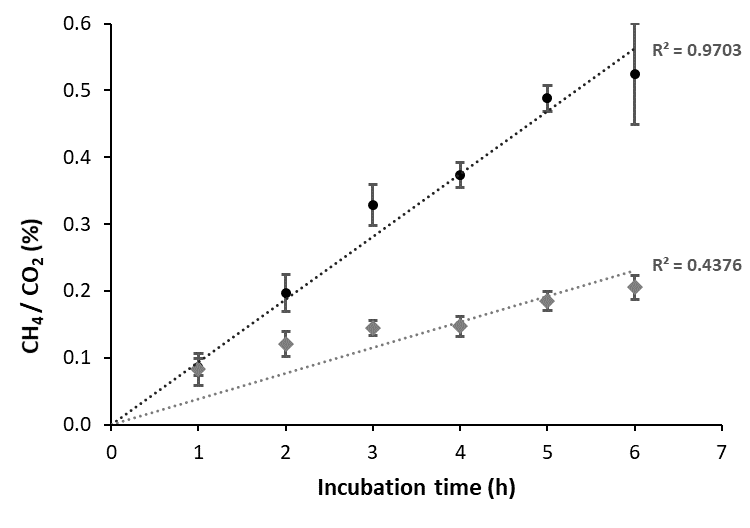


**Figure S6. Ratio of CO_2_ to CH_4_ in *M. shengliensis* ZC-1 resting cells fed with TMB.** Resting cells of *M. shengliensis* ZC-1 were incubated with 10 mM TMB for 6 h. Every hour the produced CH_4_ (black circles) and CO_2_ (grey circles) was analysed by GCMS. The *M. shengliensis* ZC-1 cells produce CO_2_ and CH_4_ at a 1:2 ratio.


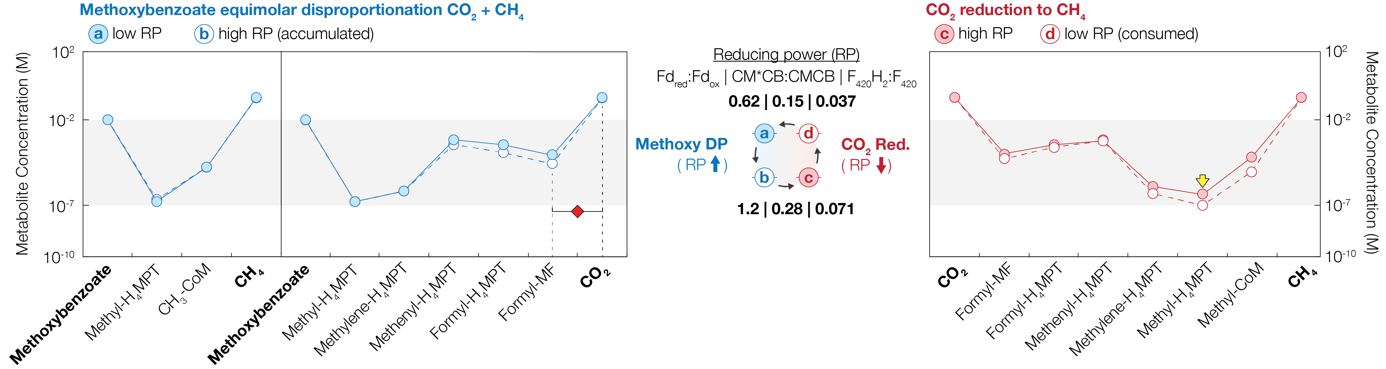


**Figure S7. Limit (quasi-equilibrium) metabolite concentrations of two-stage methoxydotrophic methanogenesis under different cytosolic reducing power concentrations.** (Left) Metabolite concentrations of methoxybenzoate disproportionation to CO_2_ and CH_4_ is shown when cytosolic reducing power (RP) concentration is low (solid blue line). At a higher RP concentration (dotted blue line), Fd_red_-driven formyl-MF reduction becomes endergonic (indicated with red diamond), so methoxybenzoate oxidation to CO_2_ becomes thermodynamically infeasible. (Right) Metabolite concentrations of CO_2_ reduction to CH_4_ is shown at the maximum cytosolic RP concentration during methoxybenzoate disproportionation (solid red line). At a lower RP concentration (dotted red line), formyl-MF, methylene-H_4_MPT, and methyl-H_4_MPT approach 1 µM (yellow arrow) and CO_2_ reduction to CH_4_ becomes kinetically infeasible. Refer to methods for specific conditions.
